# APOE2, E3, and E4 differentially modulate cellular homeostasis, cholesterol metabolism, and inflammatory response in isogenic iPSC-derived astrocytes

**DOI:** 10.1016/j.stemcr.2021.11.007

**Published:** 2021-12-16

**Authors:** Sherida M. de Leeuw, Aron W.T. Kirschner, Karina Lindner, Ruslan Rust, Vanessa Budny, Witold E. Wolski, Anne-Claude Gavin, Roger M. Nitsch, Christian Tackenberg

**Affiliations:** 1 University of Zurich, Institute for Regenerative Medicine, IREM, Wagistrasse 12, 8952 Schlieren, Switzerland; 2 Neuroscience Center Zurich, University of Zurich and ETH Zurich, Zurich, Switzerland; 3 University of Geneva, Department of Cell Physiology and Metabolism, CMU Rue Michel-Servet 1, 1211 Genève 4, Switzerland; 4 Functional Genomics Center Zurich, University of Zurich and ETH Zurich, Zurich, Switzerland

**Keywords:** iPSCs, astrocytes, isogenic, APOE, cholesterol, proteomics, lipid metabolism, inflammation, homeostasis, Aβ, Alzheimer disease

## Abstract

The apolipoprotein E4 (*APOE4*) variant is the strongest genetic risk factor for Alzheimer disease (AD), while the *APOE2* allele is protective. A major question is how different *APOE* genotypes affect the physiology of astrocytes, the main APOE-producing brain cells. Here, we differentiated human *APOE*-isogenic induced pluripotent stem cells (iPSCs) (*APOE4*, *E3*, *E2*, and *APOE* knockout [*APOE-KO*]) to functional “iAstrocytes”. Mass-spectrometry-based proteomic analysis showed genotype-dependent reductions of cholesterol and lipid metabolic and biosynthetic pathways (reduction: *APOE4* > *E3* > *E2*). Cholesterol efflux and biosynthesis were reduced in *APOE4* iAstrocytes, while subcellular localization of cholesterol in lysosomes was elevated. An increase in immunoregulatory proteomic pathways (*APOE4* > *E3* > *E2*) was accompanied by elevated cytokine release in *APOE4* cells (*APOE4* > *E3* > *E2* > *KO*). Activation of iAstrocytes exacerbated proteomic changes and cytokine secretion mostly in *APOE4* iAstrocytes, while *APOE2* and *APOE-KO* iAstrocytes were least affected. Taken together, *APOE4* iAstrocytes reveal a disease-relevant phenotype, causing dysregulated cholesterol/lipid homeostasis, increased inflammatory signaling, and reduced β-amyloid uptake, while *APOE2* iAstrocytes show opposing effects.

## Highlights

• Human astrocytes show strong proteomic differences depending on their APOE genotype
• Aβ uptake is highest in *APOE-KO* and lowest in *APOE4* astrocytes (KO > E2 > E3 > E4)
• *APOE4* astrocytes show exacerbated pro-inflammatory reactions (*APOE4* > *E3* > *E2* > KO)
• Cholesterol synthesis and efflux are reduced in *APOE4* astrocytes

## Teaser

In this article, Tackenberg and colleagues show *APOE* genotype-dependent differences in the proteomic profile of *APOE*-isogenic iPSC-derived human astrocytes. They provide evidence for a pro-inflammatory state of *APOE4* iAstrocytes, while *APOE2* and *APOE-KO* iAstrocytes show reduced inflammatory signaling. They further show the *APOE* genotype-dependent regulation of β-amyloid uptake capacity and cholesterol/lipid homeostasis and a differential reaction to inflammatory stimuli.

## Introduction

Apolipoprotein E (APOE) is part of a family of lipoprotein chaperones, binding lipoprotein complexes and facilitating their uptake through low-density lipoprotein receptors (LDLRs), or lipoprotein receptor 1 (LRP1), providing lipids and cholesterol to the cell (Kockx et al., 2018). In turn, APOE aids in cholesterol and lipid efflux from the cell by associating with lipoprotein particles upon formation (Mahley, 2016). In the brain, APOE is one of the most abundant lipoprotein chaperones, mainly expressed by astrocytes, and in low levels by microglia.

APOE mainly exists in three allelic variants, APOE2, E3, and E4, which differ in a single amino acid change at position 112 or 158: APOE2 (Cys112, Cys158), APOE3 (Cys112, Arg158), and APOE4 (Arg112, Arg158) (Liu et al., 2013). The Cys112 residue in APOE2 reduces its receptor binding capacity, congruent with the increased levels of lipoproteins in plasma, leading to hyperlipidemia III (Fernandez et al., 2019). Arg158 in APOE4 causes an ionic interaction between Arg61 and Cys255 on the lipid binding domain, inferring a structural change, leading to altered lipid binding and decreased capacity to load cholesterol.

Carrying the *APOE4* allele is the major genetic risk factor for developing sporadic Alzheimer disease (AD), where one allele confers a 3-fold increase and two alleles a 12-fold increase in risk. The *APOE2* allele is protective for AD; however, its biological role is heavily understudied and not yet understood (Michaelson, 2014). AD is the most common age-related neurodegenerative disorder and is characterized by the presence of β-amyloid (Aβ) plaques and neurofibrillary tangles. The accumulation and aggregation of Aβ, caused by reduced clearance mechanisms, plays a significant role in the progression of AD pathogenesis (Winblad et al., 2016). Early AD pathology is signified by a complex cellular phase, presenting a myriad of dysfunctions such as synapse loss, elevated immune response, defective clearance by the endolysosomal system, ER stress, and an increase in cholesteryl esters, cholesterol, and decreased levels of glycerophospholipids (De Strooper and Karran, 2016; Tackenberg et al., 2009). It has been hypothesized that these dysfunctions may be preceded by loss of homeostatic control of astrocytes (Rodríguez-Arellano et al., 2016). Considering that APOE is primarily expressed in astrocytes, a key role is implied for astrocytic APOE in the initial phase of AD pathophysiology.

We aimed to understand the fundamental role of the three different APOE isoforms (APOE2, E3, E4), in human induced pluripotent stem cell (iPSC)-derived astrocytes. After successfully deriving non-proliferative astrocytes at a resting state, we show that *APOE2*, *E3*, *E4*, and *APOE* knockout (*APOE-KO*) iAstrocytes display distinct phenotypes in homeostatic functions, cholesterol and lipid metabolism, lysosomal function, and inflammatory regulation.

## Results

### Robust differentiation of quiescent iAstrocytes from iPSC-derived neural progenitor cells

**Figure 1:**
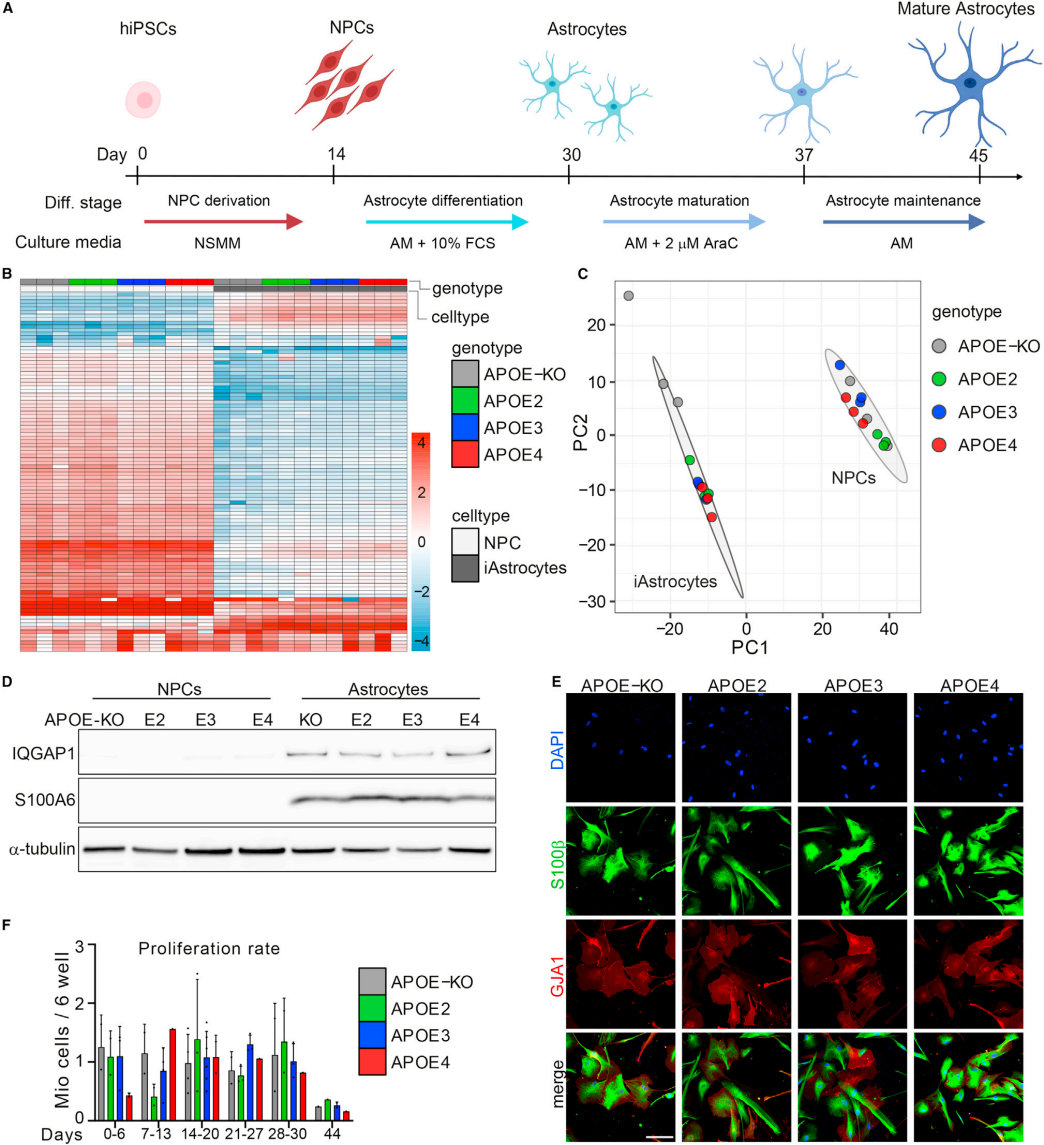
APOE-isogenic iPSCs are differentiated to iAstrocytes (A) Schematic representation of the iAstrocyte differentiation protocol. (B) Heatmap of top 100 differentially expressed proteins between NPCs and iAstrocytes selected by fold change. (C) PCA of proteomic data from NPCs and iAstrocytes of all genotypes. Three biological replicates were analyzed. (D) Western blot of astrocyte marker proteins IQGAP1 and S100A6 in *APOE*-isogenic NPCs and astrocytes. (E) Immunocytochemistry of astrocyte markers S100β and GJA1 in *APOE*-isogenic iAstrocytes at day 44. Scale bar: 150 μm. (F) Proliferation rate of *APOE*-isogenic iAstrocytes at different time points in culture. See also Figure S1.

There is currently an abundance of differentiation protocols for iPSC-astrocytes. However, the majority are laborious, require tedious cocktails of small molecules, require sorting methods presuming previous expertise, or use fetal calf serum (FCS), resulting in activated astrocytes at baseline (Zhang et al., 2016). By simply applying ScienCell Primary Astrocyte Medium (AM) to neural progenitor cells (NPCs), TCW and colleagues (TCW et al., 2017) generated functional iPSC-astrocytes in 30 days, overcoming the complicated methods currently used, albeit still using FCS. We have therefore extended this method by withdrawing FCS and adding cytosine arabinoside (AraC) to the medium at day 30, for 7 days, followed by another 7 days of AM without FCS and AraC, taking a total of 44 days and generating mature and quiescent iAstrocytes (Figures 1A and 1F).

Isogenic *APOE* iPSCs were obtained from the European Bank for Induced Pluripotent Stem Cells (EBiSC), and show mono-allelic expression of *APOE*, or *APOE* deletion (Schmid et al., 2019, 2020). Unlabeled mass spectrometry (MS)-based proteomic analysis of iAstrocytes and NPCs from four different genotypes (*APOE2*, *APOE3*, *APOE4*, and *APOE-KO*; Figure S1A) resulted in a total of 3,765 detected proteins, of which the majority, 2,432 proteins, were differentially expressed in iAstrocytes compared with NPCs (false discovery rate [FDR] <0.1), from which the 100 most differentially expressed proteins were visualized (Figure 1B). Principal component analysis (PCA) showed separate clustering of NPCs and iAstrocytes, indicating all *APOE*-isogenic lines were successfully differentiated from NPCs to iAstrocytes (Figure 1C). It should be noted that the three biological replicates of *APOE-KO* iAstrocytes are more dispersed in the PCA plot than the other genotypes, indicating higher variation in *APOE-KO* cells, and that we observed a substantially lower number of peptides and proteins in MS analysis. Since this would bias the data normalization for the *APOE-KO* samples, we omitted them from the quantitative proteomic analysis. Among the astrocyte markers significantly upregulated in iAstrocytes were calcium binding protein S100A6 and Rho-ATPase IQGAP1, which were confirmed with western blot showing robust expression in iAstrocytes, and absence in NPCs (Figure 1D). Immunocytochemical analysis of mature astrocyte markers, calcium binding protein S100β, and gap junction protein GJA1 showed all cells being positive at day 44 (Figure 1E), as well as for intermediate filament protein GFAP (Figure S1B). Compared with iAstrocytes at day 44, cells on day 30 displayed a different morphology (Figures S1C and S1D); the latter were smaller and expressed most GJA1 in the cytosol, while GJA1 was prominently expressed on the cell surface on day 44, in addition to the significant increase in cell volume demonstrated in the S100β staining. Altogether we show successful differentiation of NPCs to quiescent iAstrocytes, carrying *APOE2*, *APOE3*, *APOE4*, or *APOE-KO* alleles, showing a mature phenotype.

### Homeostatic functions show *APOE* allele-dependent decline in iAstrocytes

**Figure 2:**
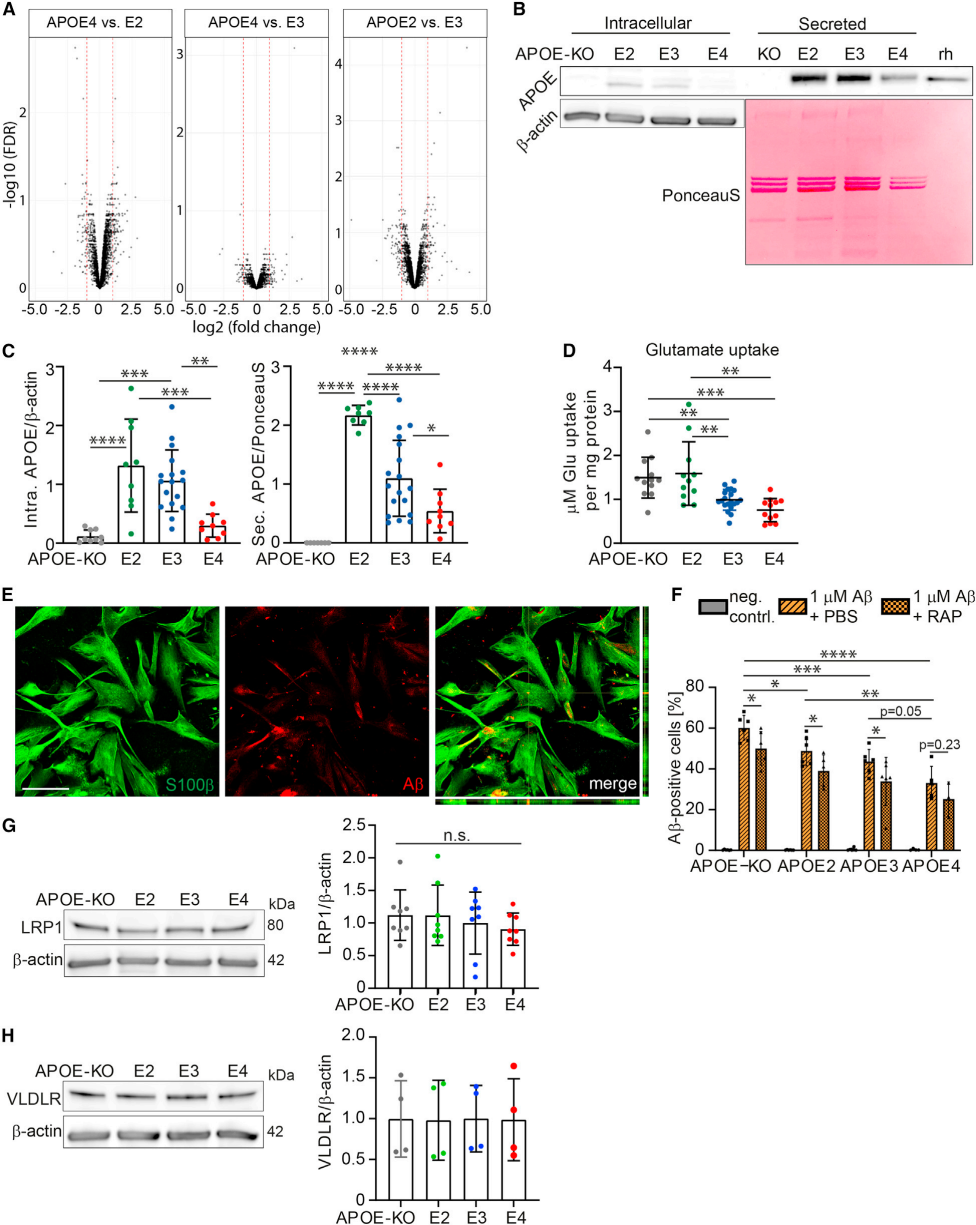
*APOE*-isogenic iAstrocytes show allele-dependent decline in homeostatic functions (A) Volcano plots of proteomic analysis comparing *APOE2* versus *APOE3*, *APOE4* versus *APOE3*, and *APOE4* versus *APOE2* iAstrocytes. Log2 of fold change is plotted against −log10 of FDR. Red lines indicate a fold change of 1. (B) Western blot of APOE in lysate (intracellular) and supernatant (secreted) of iAstrocytes. Intracellular APOE was normalized to β-actin, secreted APOE to the PonceauS signal of the respective lane. Recombinant human (rh) APOE served as control. (C) Quantification of APOE western blot bands from lysate (Intra. APOE) and supernatant (Sec. APOE). (D) Glutamate uptake assay showing the amount of glutamate taken up by *APOE*-isogenic iAstrocytes within 1 h. Data were normalized to total protein content of the respective cell lysate. (E) Representative confocal images of stained iAstrocytes (S100β), treated with 1 μM Aβ42. Scale bar: 150 μm. (F) Flow-cytometry-based Aβ42 uptake assay showing percentage Aβ-positive cells of live cells. Cells were treated with 1 μM pre-aggregated Aβ42-hilyte488 or pre-treated for 24 h with 0.2 μM LRP1 antagonist RAP. (G) Western blot and quantification of LRP1 in lysates from iAstrocytes. (H) Western blot and quantification of VLDLR in lysates from iAstrocytes. Data represent mean ± SD. ^∗^p < 0.05; ^∗∗^p < 0.01; ^∗∗∗^p < 0.001; ^∗∗∗∗^p < 0.0001, one-way ANOVA with *post hoc* Tukey's multiple comparisons test (C, D, G, and H), two-way ANOVA with *post hoc* Holm-Šídák's multiple comparisons test (F). All datapoints (n-numbers) are plotted in each bar graph (three independent experiments).

To investigate *APOE*-related phenotypes in isogenic iAstrocytes, proteomic analysis of *APOE2*, *APOE3*, and *APOE4* iAstrocytes was performed showing most differently regulated proteins (fold change above 1 or below −1) between *APOE2* and *APOE4* (173), less between *APOE2* and *APOE3* (130), and least between *APOE4* and *APOE3* (72) (Figure 2A). We analyzed APOE levels in iAstrocytes that were regulated in isoform-dependent manner (E2 > E3 > E4), and absent in *APOE-KO* iAstrocytes (Figures 2B and 2C). In line with the extracellular lipoprotein-binding function of APOE, we observed a larger amount of APOE protein in the secreted fraction, where the difference between genotypes was higher than in the lysate. APOE is known to affect many functional aspects of astrocytes, including, but not limited to, homeostatic support such as uptake of glutamate as well as uptake and degradation of Aβ, as shown in mouse models (Verghese et al., 2013).

Uptake of glutamate in iAstrocytes demonstrated an allele-dependent effect, with *APOE4* iAstrocytes taking up the lowest amount of glutamate while *APOE2* and *APOE-KO* took up the highest amounts (Figure 2D). Reduced glutamate uptake was not caused by the lower APOE levels in *APOE4* iAstrocytes, as the *APOE-KO* iAstrocytes showed similar glutamate uptake capacity to *APOE2*. As a second functional measure, we determined the ability of iAstrocytes to take up Aβ (Figure 2E). A flow-cytometry-based Aβ42 uptake assay, using pre-aggregated Hilyte488-tagged Aβ42, showed an allele-dependent trend in uptake capacity (KO > E2 > E3 ≥ E4), although differences between *APOE3* and *APOE4* did not reach significance (p = 0.054) (Figure 2F). Interestingly, *APOE-KO* cells showed the highest Aβ42 uptake capacity. Aβ42 is mostly taken up through receptor-dependent endocytosis, such as LRP1. To determine if Aβ uptake in iAstrocytes is mediated by LDLR-dependent endocytosis, we treated cells with a low concentration of LDLR family antagonist receptor-associated protein (RAP), which primarily blocked LRP1 and very-low-density lipoprotein receptor (VLDLR), in order to induce a mild reduction in Aβ uptake, resulting in a measurable difference between genotypes. Blocking LDLR family members caused a significant decrease in Aβ uptake in *APOE-KO*, E2, and E3, but not in E4 iAstrocytes (p = 0.23). Quantification of LRP1 and VLDLR showed no significant difference, albeit a minor decrease of average LRP1 levels in *APOE4* cells (Figures 2G and 2H). These results demonstrate an isoform-dependent regulation of APOE protein levels, as was previously shown for E3 and E4 *in vitro*, or in patient cerebrospinal fluid (CSF) (Lin et al., 2018; Mahley, 2016). We further show an allele-dependent effect on iAstrocyte homeostatic functions (KO = E2 > E3 > E4), where *APOE2* closely resembled *APOE-KO* iAstrocytes.

### Cholesterol and lipid metabolism show *APOE* allele-dependent regulation

**Figure 3:**
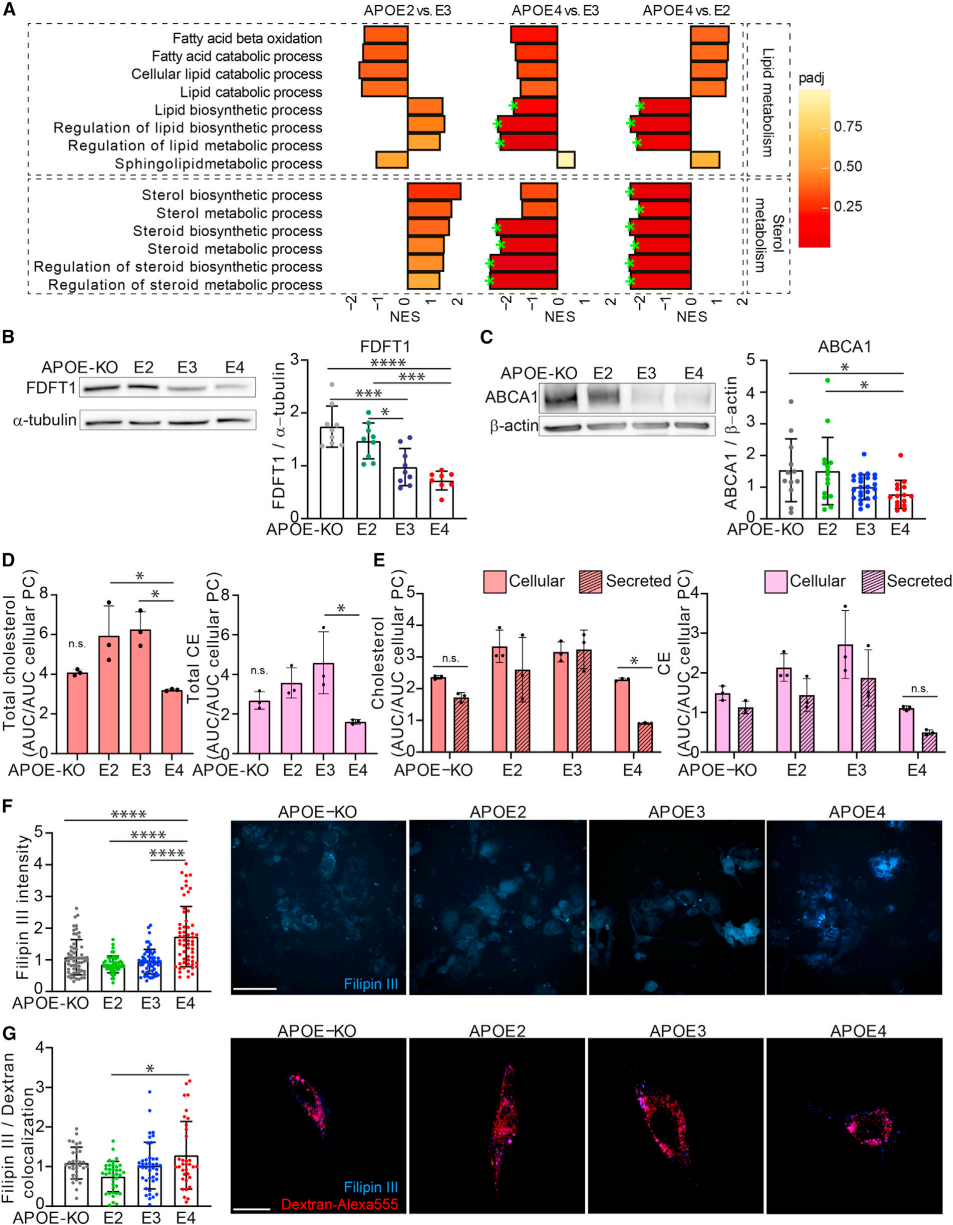
*APOE*-isogenic iAstrocytes show allele-dependent regulation of cholesterol and lipid metabolism (A) GSEA normalized enrichment scores (NESs) of protein lists ranked according to the t-statistic obtained for the contrast *APOE2* versus *APOE3*, *APOE4* versus *APOE3*, and *APOE4* versus *APOE2* iAstrocytes. NES is plotted on the x axis, with color-coded bars for the individual Gene Ontology (GO) terms, according to adjusted p value (padj). We annotated gene sets with padj <0.2 (green stars). (B) Western blot and quantification of FDFT1 in lysates from iAstrocytes, normalized to α-tubulin. (C) Western blot and quantification of ABCA1 in lysates from iAstrocytes, normalized to β-actin. (D) Total cholesterol and CE quantified with HPTLC, normalized to cellular phosphatidylcholine content. (E) Cellular and secreted cholesterol and CE quantified with HPTLC, normalized to cellular phosphatidylcholine content. (F) Bar graphs of Filipin III intensity quantification, normalized to *APOE3* intensity, with representative example images. Scale bar: 150 μm. (G) Bar graphs showing quantification of Filipin III and Dextran-Alexa 555 colocalization in iAstrocytes, normalized to *APOE3*. Representative images of Filipin III (blue) and Alexa 555 (red) overlay are displayed. N = number of images analyzed. For each plot, three experiments were conducted, 7–10 images per line were taken per experiment. Scale bar: 50 μm. Data represent mean ± SD. ^∗^p < 0.05; ^∗∗∗^p < 0.001; ^∗∗∗∗^p < 0.0001, Kruskal-Wallis test (C and G); one-way ANOVA with *post hoc* Tukey's multiple comparisons test (B, D, F); or two-way ANOVA with *post hoc* Holm-Šídák's multiple comparisons test (E). All datapoints (n numbers) are plotted in each bar graph (three independent experiments; B, C, F, and G). See also Figure S2.

Gene set enrichment analysis (GSEA) of proteomic data showed significant downregulation of pathways involved in lipid biosynthesis/metabolism and sterol biosynthesis/metabolism in *APOE4* compared with *APOE3* or *APOE2* iAstrocytes, while these were upregulated in *APOE2* compared with *APOE3* (Figure 3A). Leading-edge proteins for the analyzed pathways are listed in Table S1. One of the proteins significantly regulated was FDFT1 (squalene synthase), the first committed enzyme in the *de novo* biosynthesis pathway of cholesterol, or mevalonate pathway (Do et al., 2009). A second protein of interest was ATP-ase binding cassette 1 (ABCA1), essential for lipid and cholesterol efflux, as well as for APOE lipidation. Western blot analysis confirmed that these proteins were allele-dependently regulated (KO = E2 > E3 > E4) (Figures 3B and 3C). To investigate the functional consequences of these observations, cholesterol, cholesteryl esters (CEs), and phosphatidylethanolamines (PEs) were quantified using high-performance thin-layer chromatography (HPTLC) from cell pellet and supernatant. In line with the proteomic data, total as well as cellular and secreted cholesterol was decreased in *APOE4* compared with *APOE3* and *APOE2* iAstrocytes, while showing similar levels to *APOE-KO* (E2 = E3 > E4 = KO) (Figures 3D, S2A, and S2B), whereas cellular CEs were only significantly decreased in *APOE4* compared with E3. Cellular PE showed higher levels in E2 and E4 and triacylglycerol (TAG) displayed an isoform-dependent increase (E4 > E3 > E2 = KO). Note that TAG was not present in the secreted fraction, so cellular TAG represents the total amount. Interestingly, secreted cholesterol and CE were significantly decreased only in *APOE4* iAstrocytes (Figures S2A and S2B). CEs did not show a genotype-dependent difference in cellular versus secreted lipids (Figure 3E). The reduced secretion of cholesterol by the *APOE4* iAstrocytes could be explained by a difference in subcellular localization. We therefore visualized non-membrane-bound cholesterol by methyl-β-cyclodextrin (MβCD) treatment and subsequent Filipin III staining, as well as additional Dextran-Alexa 555 treatment, to determine lysosomal localization of cholesterol. We found that non-membrane-bound cholesterol was increased in *APOE4* iAstrocytes (E4 > E3 = E2 = KO) (Figure 3F). Further colocalization of Filipin III with Dextran-Alexa 555 was highest in *APOE4* iAstrocytes, indicating higher cholesterol levels in lysosomes of *APOE4* iAstrocytes (E4 > E2) (Figure 3G).

### Lysosomal but not endosomal function is affected by *APOE* genotype

**Figure 4:**
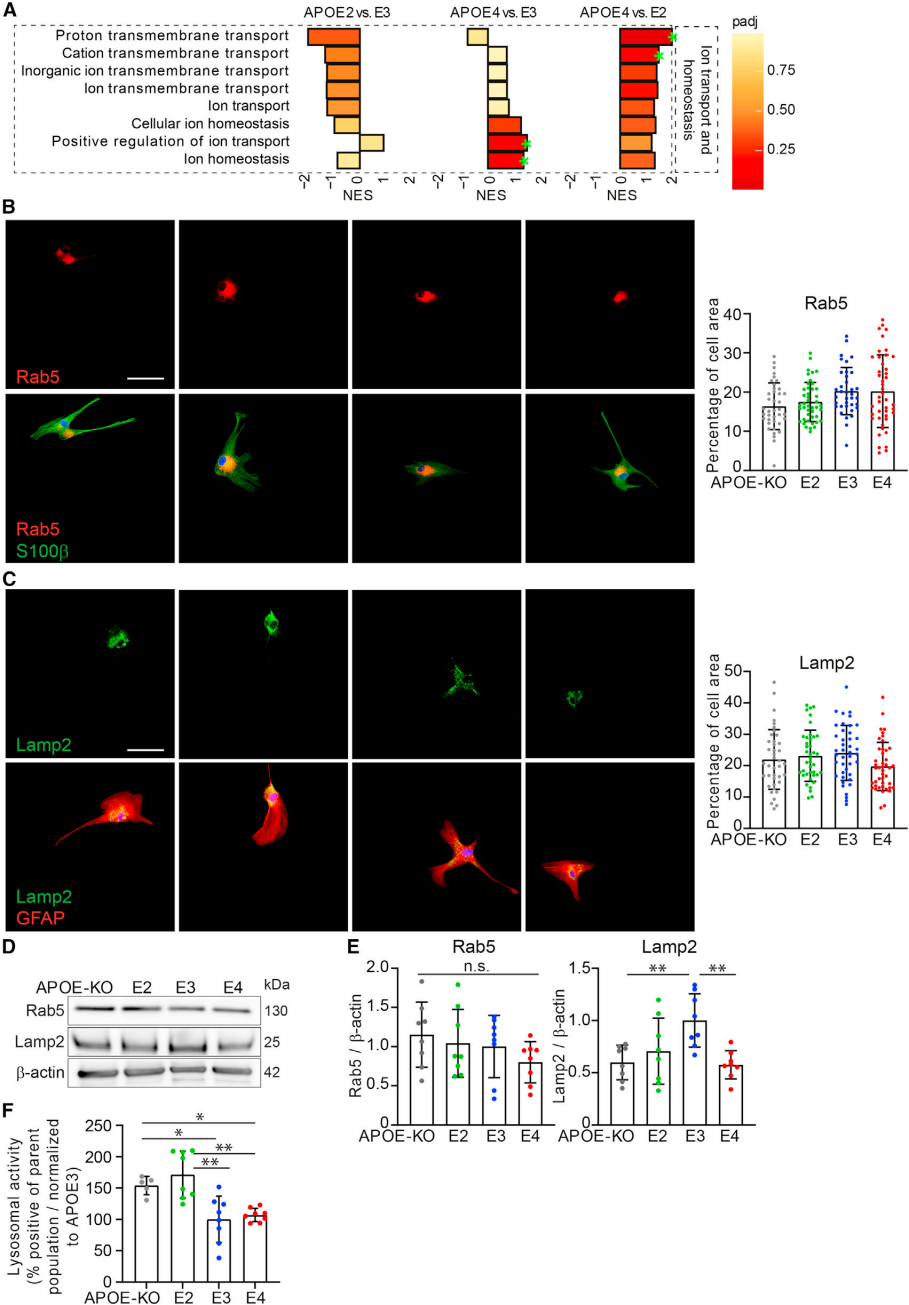
Subtle changes in lysosomal marker LAMP2 according to *APOE* genotype (A) GSEA NESs of protein lists ranked according to the t-statistic obtained for the contrast *APOE2* versus *APOE3*, *APOE4* versus *APOE3*, and *APOE4* versus *APOE2* iAstrocytes. NES is plotted on the x axis, with color-coded bars according to padj. Padj ≤0.2 was considered significant, annotated with green stars. (B) Immunocytochemical stainings of RAB5 (red, upper) and merged with S100β (green, lower). The signal is quantified and calculated as percentage of cell area. (C) Immunocytochemical stainings of LAMP2 (green, upper) and merged with GFAP (red, lower). The signal is quantified and calculated as percentage of cell area. Scale bar: 50 μm. (D) Western blot of LAMP2 and RAB5 from lysate of iAstrocytes. Scale bar: 50 μm. (E) Western blot quantification of RAB5 or LAMP2 normalized to β-actin. (F) Lysosomal activity assay in iAstrocytes, normalized to *APOE3*. Data represent mean ± SD (^∗^p < 0.05; ^∗∗^p < 0.01, one-way ANOVA with *post hoc* Tukey's multiple comparisons test). All datapoints (n-numbers) are plotted in each bar graph (three independent experiments).

To further investigate possible defects in the endolysosomal system affecting degradation of extracellular waste such as Aβ, or cholesterol trafficking, relevant pathways in our proteomic data were analyzed. Proton and cation transmembrane transport were highest in *APOE4* iAstrocytes, and lowest in E2 (Figure 4A). V-ATPase-regulated proton transport into the lysosomal lumen is crucial to maintaining the acidic pH, and defective acidification has been linked to AD or Parkinson disease (Colacurcio et al., 2018). An increase in proton and cation transmembrane transport might indicate a compensatory mechanism for underlying dysfunction. In addition, positive regulation of ion transport and ion homeostasis pathways were enriched in *APOE4* compared with *APOE3*, and other ion regulatory pathways were not significantly regulated.

We further assessed the levels of lysosomal and endosomal markers LAMP2 and RAB5. Endosomal compartments labeled with RAB5 did not show a significant difference in percentage of cell area between genotypes, and neither did RAB5 protein levels (Figures 4B, 4D, and 4E). Immunofluorescent detection of lysosomes by LAMP2 (Figure 4C) or Dextran-Alexa 555 (Figure S3) showed no difference between genotypes. However, protein levels of LAMP2 detected by western blot were significantly higher in *APOE3* compared with *APOE2*, *APOE4*, and *APOE-KO* (E3 > E2 = E4 = KO) (Figures 4D and 4E). Lysosomal activity in *APOE3* and *APOE4* was significantly lower than in APOE2 and APOE-KO cells (KO = E2 > E3 = E4) (Figure 4F). Thus, no major abnormalities in endosomal and lysosomal compartment morphology were observed, while LAMP2 protein levels and lysosomal activity were affected by *APOE* genotype.

### APOE genotype-dependent upregulation of inflammatory pathways and exacerbated cytokine release in IL-1β-treated iAstrocytes

**Figure 5:**
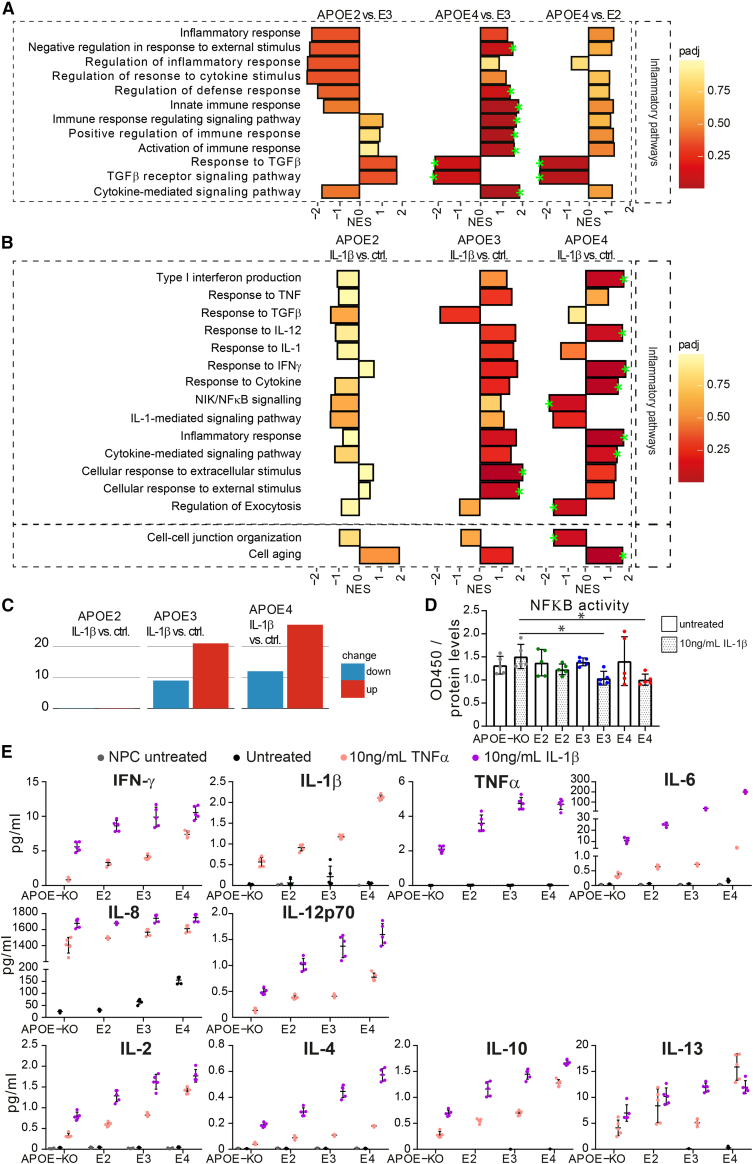
*APOE* genotype-dependent effects on inflammatory pathway expression and cytokine release (A) GSEA NESs of protein lists ranked according to the t-statistic obtained for the contrast *APOE2* versus *APOE4*, *APOE4* versus *APOE3*, and *APOE4* versus *APOE2* iAstrocytes respectively. (B) GSEA of differential protein expression of baseline versus IL-1β treated iAstrocytes (*APOE2*, *E3*, and *E4* respectively). NES is plotted on the x axis, with color-coded bars according to padj. We annotated gene sets with padj <0.2 (green stars). (C) Number of differentially expressed biological pathways determined by GSEA, with a padj <0.5. (D) NF-κB assay showing NF-κB activity in iAstrocytes at baseline (untreated) and after IL-1β treatment. (E) Levels of inflammatory cytokines secreted by NPCs at baseline (gray), iAstrocytes at baseline (black), 10 ng/mL IL-1β-treated iAstrocytes (coral), and 10 ng/mL TNF-α-treated iAstrocytes (lilac), measured with MSD. Data represent mean ± SD. All datapoints (n-numbers) are plotted in each bar graph. For the MSD graphs, data from five cell culture wells were analyzed in one experiment. The full MSD data are depicted in Table S2.

A hallmark of AD pathology is an increased inflammatory microenvironment. Reactive microglia secrete cytokines, such as interleukin (IL)-1β or tumor necrosis factor alpha (TNF-α), to induce a cellular inflammatory cascade, and activate astrocytes. To assess the role of *APOE* genotype on the inflammatory state of astrocytes, we treated iAstrocytes with IL-1β and performed proteomic analysis. At baseline, pathways involved in inflammatory regulation were highest in *APOE4* compared with E3 and E2 and lower in E2 compared with E3 (E4 > E3 > E2) (Figure 5A). When stimulated with IL-1β, the majority of these pathways were downregulated in E2, but showed enrichment in E3 and E4 (Figure 5B). On the contrary, regulation of exocytosis and nuclear factor kappa-B (NF-κB) signaling pathways were significantly downregulated in *APOE4*, but not in *E2* or *E3*. GSEA showed an allele-dependent effect in differentially regulated pathways (E4 > E3 > E2). In *APOE4* and *APOE3* iAstrocytes, inflammatory pathways were predominantly upregulated after IL-1β treatment, while the opposite was found in *APOE2* cells. Interestingly, the cell aging pathway was significantly upregulated in E4; enriched, but not significantly, in E3; and even less in E2. Lastly, cell-cell junction was significantly downregulated in E4 but only slightly in E2 and E3, indicating that an inflammatory microenvironment affects astrocytes, especially E4, on a fundamental level, from cell aging to cellular communication. Interestingly, *APOE4* iAstrocytes showed the most differentially expressed pathways (both up- and downregulated), more than *APOE3*, and *APOE2* iAstrocytes showed almost no significantly regulated pathways, indicating that *APOE4* iAstrocytes are most susceptible to pro-inflammatory stimuli (Figure 5C). Using a calorimetric NF-κB assay, we showed equal levels of NF-κB at baseline, while NF-κB activity was lower in E4 and E3 iAstrocytes after IL-1β treatment, compared with KO iAstrocytes (Figure 5D).

Release of cytokines upon IL-1β or TNF-α treatment was measured using MSD, showing a strong allele-dependent release in all cytokines (E4 > E3 > E2 > KO), except for IL-8 (Figure 5E, Table S2). This cytokine already showed detectable release at baseline, following a similar trend (E4 > E3 > E2 = KO). Interestingly, IL-6 was increased upon stimulation, but also showed detectable levels at baseline in *APOE4* iAstrocytes. We then assessed whether IL-1β treatment also affected homeostatic functions by measuring Aβ42 uptake after IL-1β treatment of the iAstrocytes, but, besides the previously shown allele-dependent degree of uptake, no significant effect of the treatment was observed (Figure S4). These data suggest that *APOE4* iAstrocytes have an inflammatory phenotype already at baseline, which is exacerbated upon activation, while *APOE2* cells display a rather anti-inflammatory or neutral phenotype, and *APOE3* represents an intermediate state.

### IL-1β treatment affects cholesterol and CE regulation

**Figure 6:**
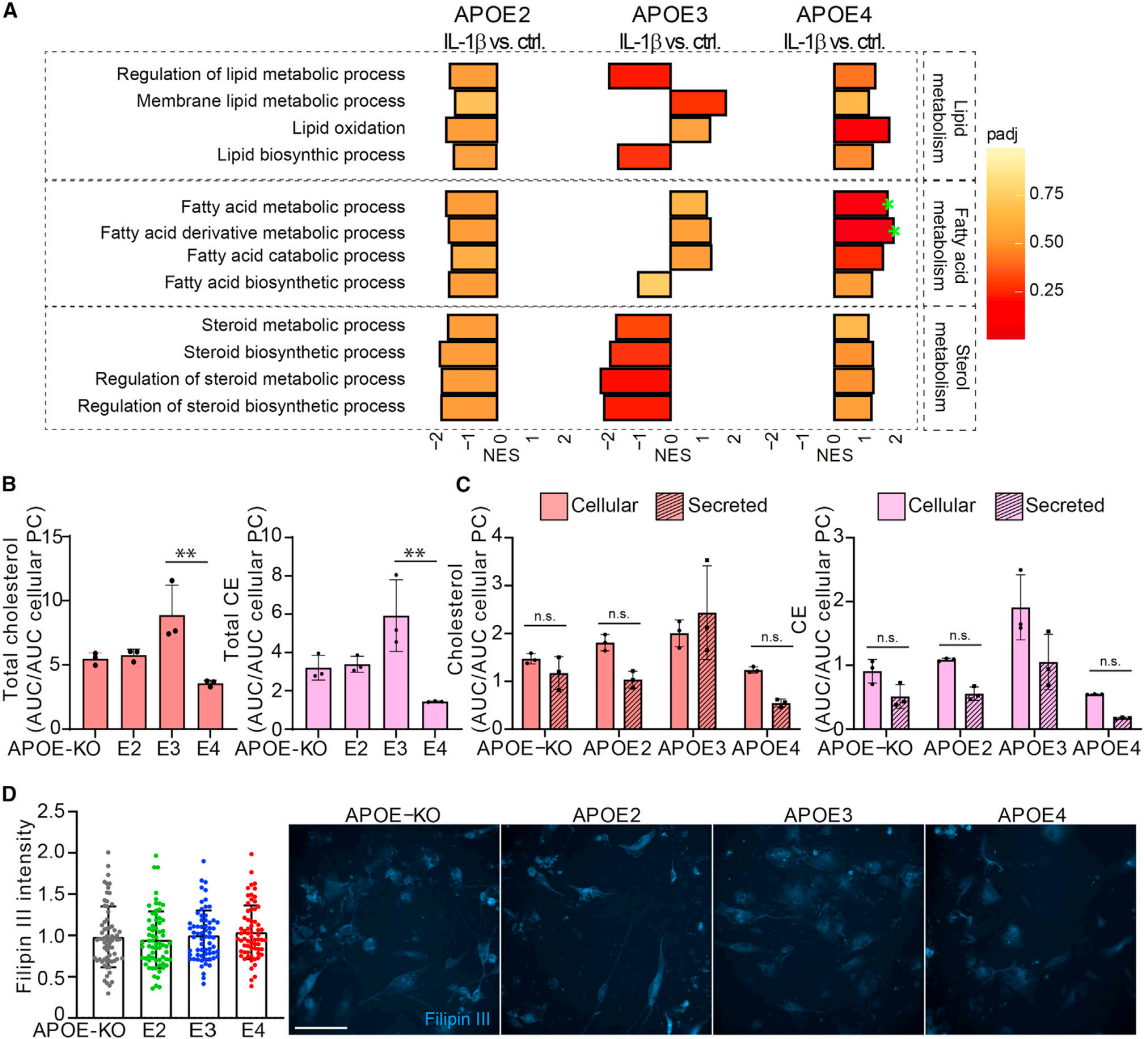
IL-1β treatment induces lipid, fatty acid, and sterol metabolism in *APOE4* iAstrocytes (A) GSEA of differential protein expression at baseline versus IL-1β-treated iAstrocytes (*APOE2*, *E3*, and *E4* respectively). NES is plotted on the x axis, with color-coded bars according to padj. Padj ≤ 0.2 was considered significant, annotated with green stars. (B) Total cholesterol and CE in IL-1β-treated iAstrocytes determined with HPTLC, normalized to cellular phosphatidylcholine. (C) Cellular and secreted cholesterol and CE in IL-1β-treated iAstrocytes determined with HPTLC, normalized to cellular phosphatidylcholine. (D) Bar graphs of Filipin III intensity quantification, normalized to *APOE3* intensity, with representative example images on the right side. Scale bar: 150 μm. Data represent mean ± SD. ^∗∗∗∗^p < 0.0001, one-way ANOVA with *post hoc* Tukey's multiple comparisons test (B and D); two-way ANOVA with *post hoc* Holm-Šídák's multiple comparisons test (C). All datapoints (n-numbers) are plotted in the bar graphs (three independent experiments).

Lastly, we assessed a potential interaction of IL-1β treatment and cholesterol, fatty acid, and lipid metabolism/biosynthesis. These pathways were mostly downregulated in *APOE4* versus *APOE2* and *APOE3* at baseline (Figure 3A), but after IL-1β stimulation they were enriched in *APOE4*, and downregulated in *APOE2*, whereas *APOE3* showed differential regulation of these pathways compared with baseline (Figure 6A). Lipid and cholesterol content was assessed after IL-1β treatment using HPTLC. Total cholesterol and CE were lowest in *APOE4* and highest in *APOE3* iAstrocytes (Figure 6B). Similar to cells at baseline, IL-1β-treated *APOE4* iAstrocytes showed the lowest levels of secreted and cellular cholesterol and CE (Figures S5A and S5B). In contrast to untreated iAstrocytes, secreted versus cellular cholesterol was not different between genotypes anymore (Figure 6C). Interestingly, IL-1β treatment induced a significant increase in cellular (Figure S5C) but not in secreted cholesterol (Figure S5D), only in *APOE3* iAstrocytes, indicating IL-1β treatment induces synthesis or uptake of cholesterol. TAGs show a genotype-dependent increase both at baseline and after activation, with lowest cellular TAG levels in *APOE-KO* and highest in *APOE4* iAstrocytes (Figures S2A and S5A). To investigate whether IL1-β treatment affected non-membrane-bound cholesterol, iAstrocytes were treated with IL-1β prior to MβCD treatment and Filipin III staining. Opposed to the increase in non-membrane-bound cholesterol at baseline, IL-1β treatment completely abolished the allele-dependent effect: levels of non-membrane-bound cholesterol were similar in IL-1β-treated iAstrocytes in all *APOE* genotypes (Figure 6D). Altogether, these data indicate a role of the inflammatory microenvironment on cholesterol regulation, with *APOE2* cells being unaffected, *APOE3* cells increasing their cholesterol content, and *APOE4* cells showing altered cholesterol regulation upon IL-1β treatment.

## Discussion

Numerous methods for deriving astrocytes from iPSCs have been successfully developed over the past years (de Leeuw and Tackenberg, 2019). With establishing an iPSC-astrocyte model to study AD-related APOE biology, it was essential that the astrocytes recapitulated a mature resting state, as they do in the human brain (Zhang et al., 2016). Even though we do use FCS in the initial stage of astrocyte differentiation, we inhibit proliferation with AraC and withdraw FCS for the last 2 weeks before analysis, slowing down cell division. Our iAstrocytes drastically changed morphology from day 30 (with FCS) to day 44 (without FCS), and show an increased membrane incorporation of gap junction protein GJA1, which is an indication of mature, adult astrocytes (Liang et al., 2020). Further, iAstrocytes showed a reduced proliferation rate, providing evidence for a further progress into a mature, resting state. In addition, iAstrocytes can be stimulated with low levels of pro-inflammatory cytokine, while being mostly non-reactive at baseline. TCW and colleagues have shown activation of astrocytes by treatment with Aβ42 peptide. Interestingly, our day 44 iAstrocytes are not susceptible to activation by Aβ42, possibly pointing out that these cells need to be primed in order to be reactive to Aβ.

### Homeostatic functions: clearance and metabolism

Astrocytes play a pivotal homeostatic role in the brain, providing neural cells with cell membrane components such as lipids and cholesterol, taking up excess of neurotransmitters at the tripartite synapse to prevent excitotoxicity, and phagocytosing cellular debris and waste, alongside microglia (Zorec et al., 2017). Many of these functions have been found to be altered or dysfunctional in AD, such as a decrease in glutamate uptake (Scott et al., 2011). We show that *APOE* has an allele-dependent effect on glutamate uptake. *APOE-KO* and *APOE2* showed similar uptake capacity, whereas *APOE3* and *APOE4* iAstrocytes displayed lowest uptake. Previous research on humanized *APOE* mice showed reduced glutamine levels, and reduced capacity to incorporate glutamate into the brain of *APOE* 4TR mice, while glutamate transporters were not significantly changed (Dumanis et al., 2013). APOE4 also interacts with insulin receptors and other transporters, relocating them to the endosomal compartment: a similar mechanism may also affect excitatory amino acid transporter (EAAT) and glutamate/aspartate transporter (GLAST) surface expression, ultimately decreasing glutamate uptake capacity in the *APOE4* iAstrocytes (Yassine and Finch, 2020).

A similar pattern was detected in Aβ42 uptake (KO > E2 > E3 ≥ E4), which is likely facilitated by APOE-bound Aβ42 clearance; a complex formation that is genotype dependent (E2 > E3 > E4) (Aleshkov et al., 1997). Interestingly, *APOE-KO* iAstrocytes were most proficient in taking up Aβ42, probably through direct lipoprotein receptor uptake of Aβ42 (Basak et al., 2012). APOE-dependent Aβ42 uptake may be the primary mode of degradation, even though an APOE-independent route, as seen in *APOE-KO* iAstrocytes, might be more efficient. Aβ42 uptake was reduced by an LRP1/VLDLR antagonist in all genotypes except *APOE4*. However, LRP1 and VLDLR did not show a genotype-dependent difference in total protein level.

Altogether we provide evidence that the presence of the *APOE4* allele induces a gain of (toxic) effect. *APOE2* and *APOE-KO* iAstrocytes show a similar phenotype, indicating differential homeostatic mechanisms by different APOE isoforms, or lack thereof, in human iAstrocytes. The *APOE* allele-dependent effect on glutamate uptake and LRP1-dependent Aβ42 uptake can provide a mechanism explaining the biological role of APOE in homeostatic functions such as regulation of glutamate levels, but also pathological events such as extracellular Aβ42 buildup.

### Lipid and cholesterol metabolism and the endolysosomal system

GSEA of the proteomic dataset revealed downregulation of lipid/sterol metabolism and biosynthesis in *APOE4* compared with *APOE3* and *APOE2* iAstrocytes, whereas lipid catabolic processes show opposite effects. To functionally validate these findings, we performed in-depth HPTLC-based lipid profiling. Triacylglycerols are subject to lipid catabolism, where they are broken down into glycerol and fatty acids, followed by fatty acid β-oxidation (Edwards and Mohiuddin, 2021), another pathway differentially regulated by the *APOE* genotype. Our HPTLC results showed an allele-dependent increase in cellular TAGs, the main component of lipid droplets (LDs), indicating potential LD accumulation, which is in agreement with a study showing increased LDs in *APOE4* compared with *APOE3* astrocytes (Sienski et al., 2021). This may lead to an imbalance in lipid metabolism/biosynthesis and catabolism, which are counter-regulated in *APOE2* and *APOE4* iAstrocytes. These results are in line with a recent study showing increased lipid content and decreased fatty acid oxidation in *APOE4* mouse astrocytes (Qi et al., 2021).

Cholesterol and CEs showed an allele-dependent decrease in cellular content (E2 = E3 > E4 = KO) and efflux (E2 = E3 = KO > E4), in line with our proteomic findings. The first committed enzyme in *de novo* biosynthesis of cholesterol, squalene synthase (FDFT1), showed very strong allele-dependent downregulation (E4 > E3 > E2 = KO), indicating that indeed these cells have a decreased capacity to produce cholesterol (Do et al., 2009). At the same time, ABCA1 also showed allele-dependent expression levels (KO = E2 > E3 > E4). This protein is vital in facilitating cholesterol efflux and is upregulated in a positive feedback loop via liver X receptor (LXR), or LXR-independent cholesterol efflux, as well as APOE lipidation and production of cholesterol-rich high-density lipoprotein (HDL) particles (Fan et al., 2018). Further, the excess TAG binding and occupying APOE4 can leave the lipoprotein unable to aid in cholesterol efflux. Even though an APOE-independent mode of efflux might be possible, *APOE2* and *APOE3* iAstrocytes show significantly higher levels of cellular cholesterol and CEs, indicating that E2 or E3 is necessary to maintain adequate levels of cellular cholesterol and CEs. Another study showed that APOE4 and ABCA1 co-aggregate, reducing ABCA1 recycling to the membrane (Rawat et al., 2019). We further show that total levels of ABCA1 also are decreased in *APOE4* iAstrocytes, suggesting it is not solely a mechanistic dysfunction but decreased protein level altogether.

We showed an increase in non-membrane-bound cholesterol in *APOE4* iAstrocytes (E4 > E3 = E2 = KO), which is in agreement with previous observations of increased cholesterol in *APOE4* compared with *APOE3* astrocytes (Lin et al., 2018; Sienski et al., 2021). The increase in non-membrane-bound cholesterol is in line with the decrease in cholesterol efflux in *APOE4* cells; membrane-bound cholesterol can bind to HDL particles and be secreted, and APOE-mediated efflux may be ineffective in *APOE4* due to the lack of ABCA1. APOE4-bound cholesterol, taken up by the cell, is released from the receptor in the late endosome or lysosome, and aggregation of APOE4 has been shown to cause endosomal congestion and dysfunction (Narayan et al., 2020; Nuriel et al., 2017; Rawat et al., 2019). We observed an increased colocalization of non-membrane-bound cholesterol to lysosomes (E4 > E3 = KO > E2). Although we did not detect significant changes in RAB5 protein levels or cell area covered, it does not exclude the presence of functional changes in endosomes or it may require prior activation of the astrocytes to detect endosomal deficits, which would thus not be present in our cells at baseline. However, we did observe differences in lysosomal LAMP2 protein levels (E3 > E2 = E4 = KO). It is known that a decrease in LAMP2 levels causes a lack of lysosomal trafficking and decrease in autophagic flux (Cui et al., 2020). Remarkably, *APOE3* iAstrocytes displayed the highest levels, while other genotypes showed similar lower levels. We further show that lysosomal activity in *APOE2* and *APOE-KO* was significantly higher than in *APOE3* and *APOE4* iAstrocytes. We therefore hypothesize that the increased LAMP2 levels in *APOE3* iAstrocytes may be a compensatory mechanism for the lower lysosomal activity, relative to *APOE2* and *APOE-KO*, while *APOE4* shows a lack of compensation. This indicates that APOE4 has the strongest detrimental effect on the functionality of the lysosomal system.

### Cholesterol metabolism and inflammatory regulation

Immune regulatory pathways in iAstrocytes at baseline were negatively enriched in *APOE2* versus E3, highly positively enriched in *APOE4* versus E3, and moderately enriched in *APOE4* versus *E2*. Two pathways related to TGF-β signaling were highly downregulated *APOE4*, in line with a study showing dysfunction in TGF-β signaling in models of early AD and in human AD brain (Tesseur et al., 2006), likely connected to its neuroprotective role. Additionally, more pathways were affected in total (both up and down) in *APOE4* iAstrocytes than in *APOE3* and *APOE2*, indicating that *APOE4* iAstrocytes are more susceptible to inflammatory stimuli. Interestingly, two of the pathways downregulated in *APOE4* iAstrocytes were NIK/NF-κB- and IL-1-mediated signaling, which were not significantly regulated in *APOE3* or E2. Although unexpected, the NF-κB activity was reduced in IL-1β-treated E4 iAstrocytes compared with KO, which supports our proteomic data. However, IL-1β-treated E3 iAstrocytes also had lower NF-κB activity, while no changes in NF-κB proteomic pathways were observed. Many studies show an increase in NF-κB in the AD brain (Liddelow and Barres, 2017). However, we also cannot exclude that a short, transient increase in NF-κB activity is enough to induce an inflammatory phenotype, as suggested previously (Liddelow and Barres, 2017), and that NF-κB activity drops again afterward.

Using MSD analysis, we observed a strong allele-dependent increase in inflammatory cytokine release upon iAstrocyte activation (E4 > E3 > E2 > KO), and even IL-6 and IL-8 release from *APOE4* at baseline. This indicates *APOE4* iAstrocytes already have an increased inflammatory phenotype, which is exacerbated in reactive iAstrocytes. This is in line with research showing that chronic inflammation is associated with an earlier onset of AD in *APOE4* carriers. Our findings of an inflammatory phenotype in resting *APOE4* iAstrocytes could explain the mechanistic link between chronic inflammation and AD pathogenesis in *APOE4* carriers (Tao et al., 2018).

Activation of iAstrocytes also resulted in an increase in pathways classified as lipid, fatty acid, and cholesterol metabolic processes in *APOE4* iAstrocytes, while the opposite effect was seen in *APOE2* iAstrocytes. *APOE3* cells showed a decrease in sterol metabolic pathways upon activation, while lipid and fatty acid metabolism were not regulated uniformly. These results oppose the findings at baseline. The significant difference between intracellular and secreted cholesterol in *APOE4* iAstrocytes was abolished after IL-1β treatment but had no effect on CE or PE, indicating that IL-1β treatment specifically affects cholesterol homeostasis. It has been shown that cytokine stimulation can regulate cholesterol synthesis by activating SREBP1 (Gierens et al., 2000). This transcription factor in turn induces *de novo* biosynthesis of cholesterol and increases the expression of LRP1 or VLDLR to increase cholesterol uptake. However, cellular cholesterol did not show an increase upon IL-1β treatment in *APOE4* iAstrocytes. Cholesterol biosynthesis and lipid efflux machinery are regulated via LXR and retinoid X receptor (RXR) complex activation; when intracellular cholesterol is increased, LXR/RXR increases ABCA1 expression to induce lipid efflux, APOE expression, and inhibition of SREBP activation (Shimano and Sato, 2017). Our results show an allele-dependent regulation of cellular cholesterol (E4 = KO > E3 < E2), with more cholesterol being translocated into the lysosomes in *APOE4* iAstrocytes, which could lead to a lower degree of LXR activation. LXRs also inhibit NF-κB signaling (Bi et al., 2016), but this pathway is shown to be reduced in *APOE4* iAstrocytes, indicative of an inherent dysfunction in the signaling complex. The LXR/RXR complex is in turn linked to transcription factor peroxisome proliferator-activated receptor gamma (PPARγ), which can also induce ABCA1 and APOE expression. In line with our results, PPARγ signaling was decreased in *APOE4* transgenic mouse brain, and increased in *APOE2* (Wu et al., 2018). Furthermore, genetic variability of LXR may contribute to the risk for AD, in addition to the ability of LXR agonists to attenuate Aβ pathology (Kang and Rivest, 2012). Hence there may be deficits in the metabolically linked PPARγ and LXR/RXR complex, rendering them less potent to induce cholesterol upregulation upon IL-1β activation in *APOE4* iAstrocytes, as seen in our results. However, LXR/RXR are likely not entirely dysfunctional as cellular/secreted cholesterol is not significantly different after IL-1β treatment in contrast to baseline, suggesting residual LXR activity allowing for the relative increase in efflux of cholesterol.

In addition, IL-1β treatment abolished the allele-dependent non-membrane-bound increase in cholesterol load that was observed at baseline by Filipin III staining. Considering the significant increase in cellular *APOE3* cholesterol (and a non-significant increase in *APOE2*) upon IL-1β treatment, which was not present in *APOE4*, we conclude that the effect of IL-1β seen is due to an increase of total cholesterol in the *APOE2* and *E3* iAstrocytes, but not in *APOE4*.

Together, we show that *APOE2*, *E3*, *E4*, and *KO* iAstrocytes show distinct phenotypes in homeostatic functions, cholesterol and lipid metabolism, lysosomal function, and inflammatory regulation. While several cellular functions, such as glutamate uptake, seem to display a clear allele-dependent decrease in capacity, the interplay between cholesterol metabolism and immune regulation indicates that it is not a mere decrease of function in *APOE4* cells: *APOE2*, *APOE3*, and *APOE4* iAstrocytes may utilize different mechanisms altogether. Further mechanistic insights into the difference in *APOE2* and E4 iAstrocytes are imperative to continue the understanding of fundamental APOE biology, which simultaneously proves its inherent relevance in AD. Our human isogenic mature iAstrocyte cell model represents a powerful tool to further address these open questions.

## Experimental procedures

### Cells

iPSC lines were purchased from EBiSC (BIONi010-C-2, -C-3, C-4, and -C-6), harboring a single *APOE2*, *APOE3*, or *APOE4* allele generated using CRISPR, or an *APOE-KO* generated by an insertion-deletion mutation in exon 2 (Schmid et al., 2019, 2020). IPSC lines were used as obtained without further modification. Data shown in this manuscript for *APOE-KO* cells are derived from the BIONi010-C-3 iPSC line, data for *APOE2* cells are derived from the BIONi010-C-6 iPSC line, data for *APOE3* cells from the BIONi010-C-2 iPSC line, or *APOE4* data from the BIONi010-C-4 iPSC line.

### iPSC differentiation to NPCs

iPSCs were maintained on Vitronectin (StemCell Technologies)-coated plates in TesRE8 medium (StemCell Technologies) and passaged using ReLeSR (StemCell Technologies). iPSCs and subsequent cell types were frozen in BAMBANKER serum-free cryopreservation medium (Wako Chemicals). NPCs were derived as described (Sancho-Martinez et al., 2016) with minor adjustments. iPSCs were dissociated with Accutase (Sigma-Aldrich), and passaged onto Vitronectin-coated plates, at 80,000 cells/well of a 12-well plate, in TesRE8 medium plus 2 μM thiazovivin. Within 24 h the cells were washed with PBS (Sigma-Aldrich)and changed with TesRE8 medium. On day 0, cells should be approximately 20% confluent, and were washed with PBS and changed with Neural Induction Medium 1 (NIM-1: 50% advanced DMEM/F12 [Invitrogen], 50% Neurobasal [Invitrogen], 1× N2 [Gibco], 1× B27 [Gibco], 2 mM GlutaMAX [Gibco] and 10 ng/mL hLIF [Peprotech], 4 mM CHIR99021 [Miltenyi], 3 mM SB431542 [Miltenyi], 2 mM Dorsomorphin [Miltenyi], and 0.1 mM Compound E [StemCell Technologies]). Medium was changed daily for 2 days, then cells were washed with PBS and switched to Neural Induction Medium 2 (NIM-2: 50% Advanced DMEM/F12, 50% Neurobasal, 1× N2, 1× B27, 2 mM GlutaMAX, 10 ng/mL hLIF, 4 mM CHIR99021, 3 mM SB431542, and 0.1 mM Compound E). After another 4 days, cells were approximately 80% confluent and passaged at 1:4 from 12-well plates to 15 μg/mL poly-L-ornithine (Sigma-Aldrich) plus 10 μg/mL laminin (Sigma-Aldrich)-coated six-well plates, in neural stem cell maintenance medium (NSMM: 50% Advanced DMEM/F12, 50% Neurobasal, 1× N2, 1× B27, 2 mM GlutaMAX, 10 ng/mL hLIF, 3 mM CHIR99021, and 2 mM SB431542). Medium was changed every day, and NPCs were kept at high density: passaged once 90%–100% confluent, approximately twice a week. Two micromolar Thiazovivin was added to the medium for the first seven passages and, after five passages, 5 ng/mL FGF (Thermo Fisher) plus 5 ng/mL epidermal growth factor (EGF) (Thermo Fisher) was added to NSMM. For assays, NPCs were plated at 200,000 cells per well of a 24-well plate.

### NPC differentiation to iAstrocytes

Between passages 1 and 4, NPCs were differentiated to astrocytes by the application of primary AM (ScienCell: 1801, AM [1801-b], 2% FCS [0010]) for 30 days according to TCW et al. (2017). NPCs were plated on 1 μg/mL Fibronectin (Sigma-Aldrich)-coated plates, at 135,000 cells/well of a six-well plate in NSMM. After 24 h, the cells were washed with PBS and changed to AM. Medium was changed every other day, and cells were split at initial density once 80%–100% confluent. To obtain mature, non-proliferative cells, astrocytes were replated on day 30 to 350,000 cells/well of a six-well plate, in AM without FCS + 2μM AraC (AM+). Medium was changed every other day; cells were not actively proliferating anymore so no passaging was needed. At day 37, the astrocytes were washed with PBS and medium replaced with AM without FCS and without AraC (AM−). Astrocytes were kept in AM− until day 44 and subsequently plated for experiments accordingly: μ-Slide Angiogenesis slides (Ibidi), 10,000 cells/well; 96-well plates, 15,000 cells/well; 24-well plates plus coverslips, 80,000 cells/well; 24-well plates, 200,000 cells/well; and six-well plates, 350,000 cells/well. Cells were always plated 24 h prior to treatment or assay.

### Immunocytochemistry

Astrocytes were grown on coverslips for 1–2 days and fixed with 4% paraformaldehyde (Electron Microscopy Science) in PBS plus 4% sucrose (Sigma-Aldrich) for 20 min at room temperature (RT), and washed three times with PBS. Cells were blocked in 10% donkey serum with 0.1% Triton X-100 in PBS for 1 h at RT, and subsequently incubated with primary antibody in 3% donkey serum with 0.1% Triton X-100 in PBS overnight at 4°C. Primary antibodies were used accordingly: S100β (Sigma, S2532, 1:200), GJA1 (Abcam, ab235585, 1:200), GFAP (Sigma, G9269, 1:200), RAB5 (Abcam, ab218624, 1:1,000), LAMP2 (BioLegend, 354302, 1:100), Aβ 1-42 (Invitrogen, 44-344, 1:1,000). Cells were washed three times with PBS and incubated with the respective secondary antibody at 1:500 in 3% donkey serum with 0.1% Triton X-100 in PBS for 2 h at RT (anti-rabbit Alexa Fluor 568 and anti-mouse Alexa 488, both from donkey, Jackson Immunoresearch). Cells were washed once and incubated with 0.2 μg/mL DAPI (4′,6-diamidino-2-phenylindole, Sigma-Aldrich) for 10 min at RT. Cells were washed twice with PBS before mounting with Mowiol 4-88 (Sigma-Aldrich).

### Meso-scale discovery cytokine measurement

Astrocytes or NPCs were plated on 24-wells plates 24 h prior to treatment. Both cell types were incubated with medium only, additionally astrocytes were treated with 10 ng/mL IL-1β or TNF-α (StemCell Technologies), for 24 h. Supernatant was transferred to fresh Eppendorf tubes, and cells were harvested for protein samples and used in label-free MS. Supernatant was centrifuged at 21,000 × *g* for 5 min at 4°C, and diluted at 1:4 in Diluent 2 from the MSD MULTI-Spot Assay system, pro-inflammatory panel 1 (human) kit. With this kit, 10 cytokines were measured according to protocol: IFN-γ, IL-1β, IL-2, IL-4, IL-6, IL-8, IL-10, IL-12p70, IL-13, and TNF-α.

### Dextran-Alexa 555 and Filipin III staining

Astrocytes were seeded onto μ-Slide Angiogenesis slides, and were additionally treated with Dextran-Alexa 555 (Thermo Fisher) if needed, otherwise cells were immediately used for Filipin III staining. Incubation with 0.2 mg/mL Dextran-Alexa 555 was performed overnight, and followed by a 14-h chase period (changed to medium only to allow Dextran localization to the lysosomes). Astrocytes were then treated with 10 mM methyl-β-cyclodextrin for 30 min, followed by fixation and treatment with the Cholesterol Assay Kit (Abcam), according to protocol with minor adjustments. After fixation, astrocytes were incubated with Filipin III for 2 h, followed by washing steps accordingly. Filipin images alone were acquired with fluorescence microscopy, Dextra-Alexa 555, and Filipin double labeling imaged with confocal microscopy. For the colocalization analysis, the area of Dextran staining was calculated of the total area of Fillipin III signal, normalized to *APOE3*.

### Immunofluorescence imaging and image processing

Confocal images were acquired with a Leica SP8 microscope, equipped with an HC PL APO CS2 20× objective (NA 0.75), using the Leica Application Suite X software. Fluorescent images were acquired with a Zeiss Axio Observer 7 equipped with an ORCA-flash4.9 camera and Plan-Apochromat 20× objective (NA 0.8), using the Zen 3.1 pro software. Image analysis and post processing was done in ImageJ or Imaris (Oxford Instruments).

### Statistical analysis

Data are presented as mean plus or minus standard deviation (SD). All datapoints (n-numbers) are plotted individually in each bar graph. Statistical analysis was performed with GraphPad Prism (GraphPad Software) and RStudio. Proteomic datasets were analyzed and visualized using edgeR (Robinson et al., 2010). Experiments were performed at least in triplicate, and a normality and lognormality test was performed if outliers were identified with the ROUT method (Q = 1%). Significance was tested using Shapiro-Wilk test for normal distribution followed by Kruskal-Wallis test, with *post hoc* Dunn's multiple comparisons test, or a one-way followed by *post hoc* Tukey's multiple comparisons testing, or two-way ANOVA with *post hoc* Holm-Šídák's multiple comparisons test. The statistical tests used in the different experiments are annotated in the respective figure legends.

## Author contributions

Conceptualization, S.M.d.L. and C.T.; methodology, S.M.d.L. and C.T.; software, R.R. and W.W.; validation, S.M.d.L., A.W.T.K. and K.L.; formal analysis, S.M.d.L., A.W.T.K., K.L., R.R., W.W., and C.T.; investigation, S.M.d.L., A.W.T.K., K.L., and V.B.; resources, A.C.G. and R.M.N.; writing, S.M.d.L. and C.T.; visualization, S.M.d.L., R.R., W.W., and C.T.; supervision, A.-C.G. and C.T.; project administration, C.T.; funding acquisition, C.T.

## Declarartion of interests

The authors declare no competing interests.

## Supplemental information

Document S1. Supplemental experimental procedures and Figures S1–S5

Table S1. NES and leading-edge proteins of analyzed pathways

Table S2. Statistical analysis of MSD

Document S2. Article plus supplemental information
