# APOE2, E3, and E4 differentially modulate cellular homeostasis, cholesterol metabolism, and inflammatory response in isogenic iPSC-derived astrocytes

**DOI:** 10.1016/j.stemcr.2022.03.018

**Published:** 2022-05-10

**Authors:** Sherida M. de Leeuw, Aron W.T. Kirschner, Karina Lindner, Ruslan Rust, Vanessa Budny, Witold E. Wolski, Anne-Claude Gavin, Roger M. Nitsch, Christian Tackenberg

## Main text

(Stem Cell Reports *17*, 110–126; January 11, 2022)

In the initial version of this article, there was an error in the merged image for *APOE3* in Figure 1E. While all individual images for S100β and GJA1 were displayed correctly, we accidentally merged *APOE3* GJA1 with *APOE2* S100β (and not with the S100β image of APOE3). However, this error did not affect the figure’s meaning or conclusion. The correct merged GJA1/S100β staining for *APOE3* iAstrocytes has now been included in the article online and below. No correction of the text or figure legend was necessary.

**Figure:**
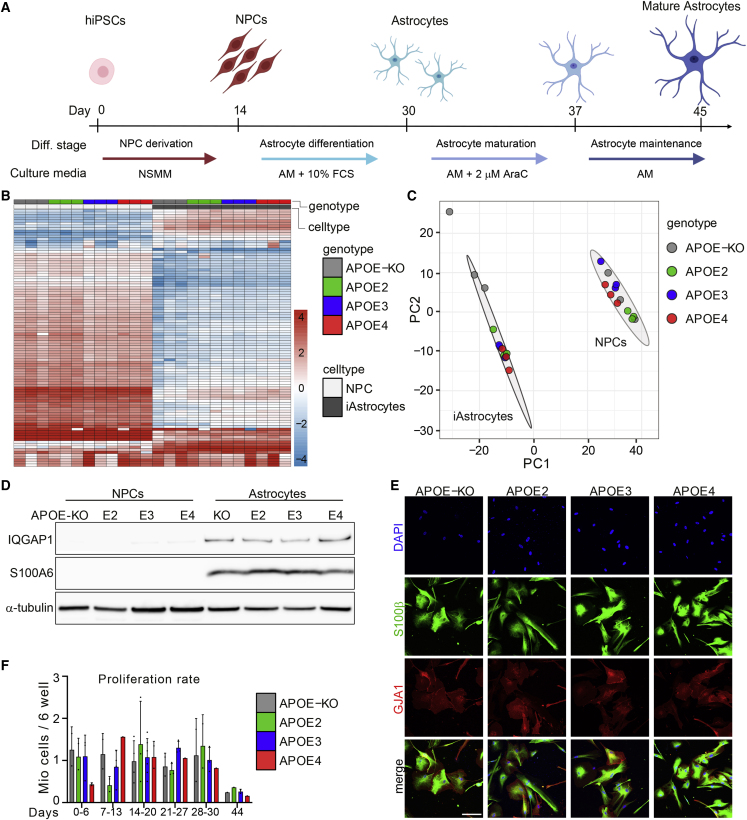
Figure 1. APOE-isogenic iPSCs are differentiated to iAstrocytes (corrected)

**Figure:**
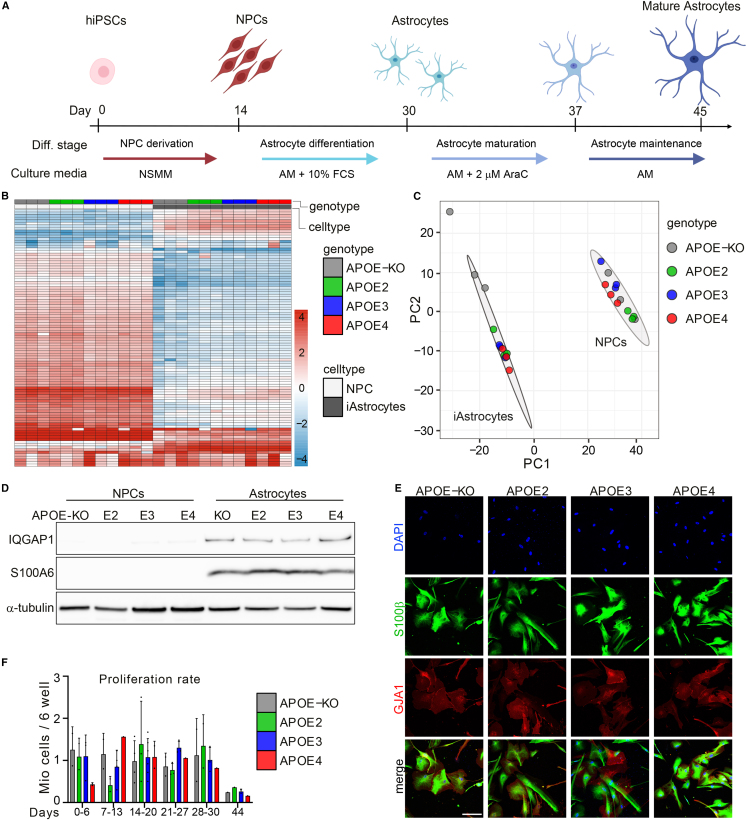
Figure 1. APOE-isogenic iPSCs are differentiated to iAstrocytes (original)

