# Supplementary material for: APOE2, E3, and E4 differentially modulate cellular homeostasis, cholesterol metabolism, and inflammatory response in isogenic iPSC-derived astrocytes
Source: Stem Cell Reports. 2021 Dec 16;17(1):110–26. doi: 10.1016/j.stemcr.2021.11.007 (PMC8758949; doi:10.1016/j.stemcr.2021.11.007)
Supplement: Document S1. Supplemental experimental procedures and Figures S1–S5 [file mmc1.pdf]

Stem Cell Reports, Volume *17*

Supplemental Information

APOE2, E3, and E4 differentially modulate cellular homeostasis, cholesterol metabolism, and inflammatory response in isogenic iPSC-derived astrocytes

Sherida M. de Leeuw, Aron W.T. Kirschner, Karina Lindner, Ruslan Rust, Vanessa Budny, Witold E. Wolski, Anne-Claude Gavin, Roger M. Nitsch, and Christian Tackenberg

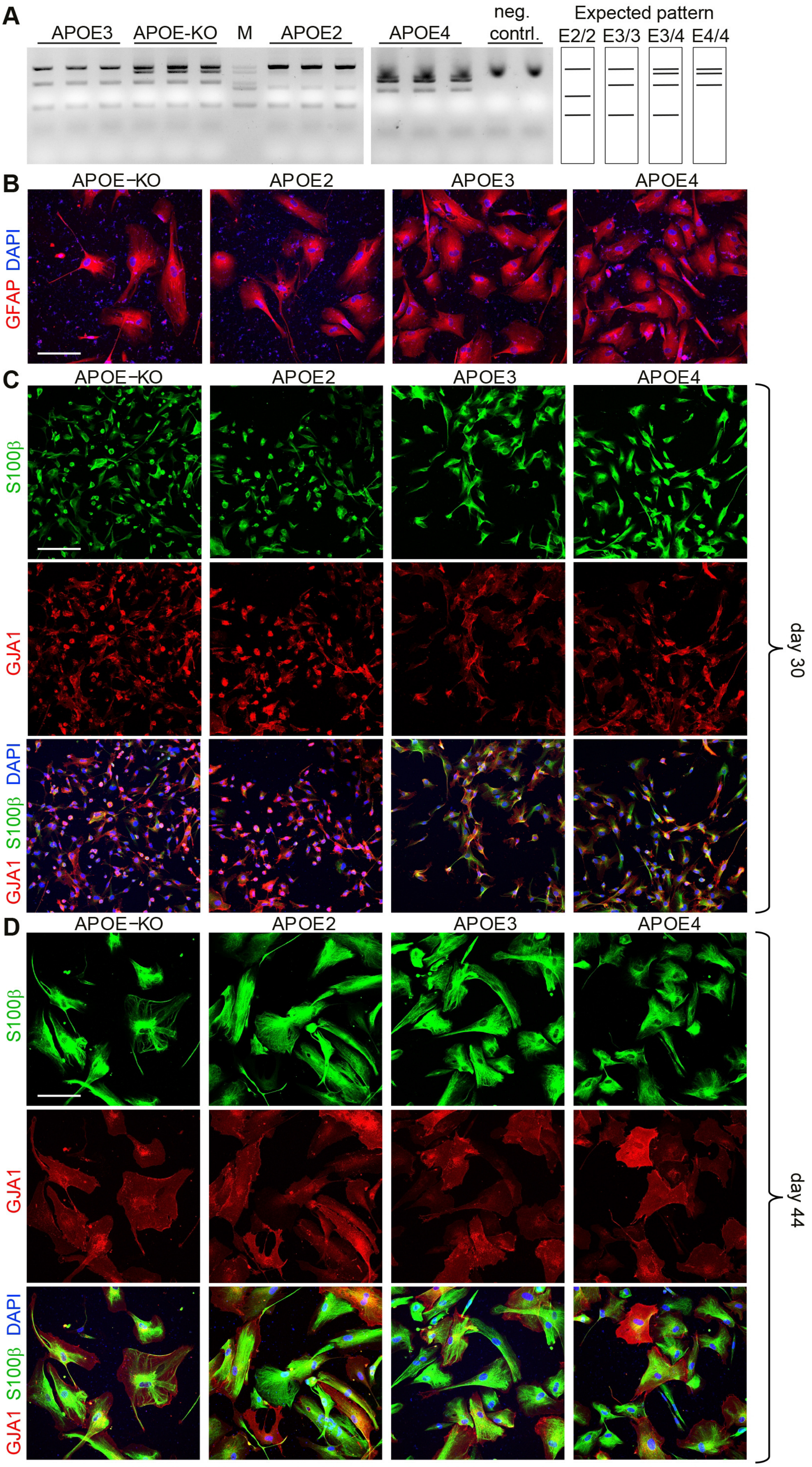

Figure S1. Genotyping and immunocytochemical characterization of APOE-isogenic iAstrocytes, related to Figure 1

A: Agarose gel of genotyping PCR using EzWay Direct APOE Genotyping Kit with a schematic representation of the expected band pattern. **B:** Confocal images of iAstrocytes at day 44, stained with antibody against astrocyte marker GFAP. Scale bar: 150µm. **C, D:** Confocal images of iAstrocytes at day 30 **(C)** or day 44 **(D)**, stained with antibody against astrocyte markers S100β and GJA1. Scale bar: 150µm.

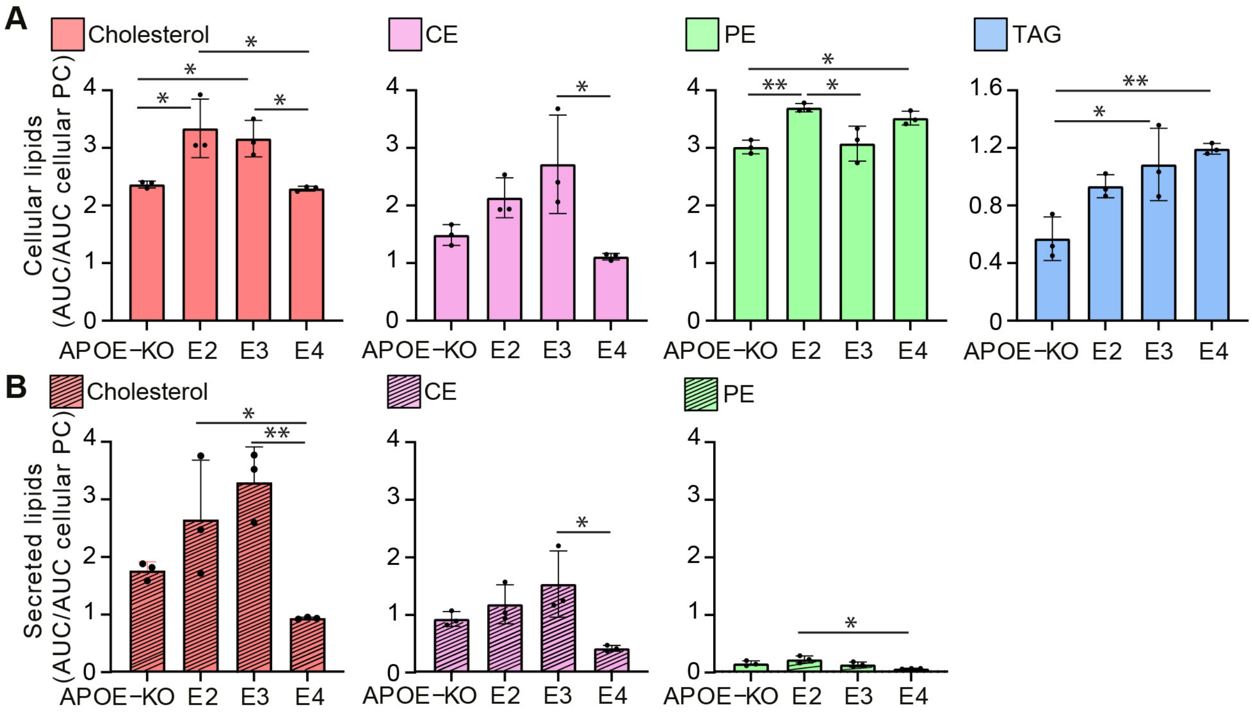

Figure S2. Cholesterol, CE and lipid analysis of the cellular and secreted fractions of iAstrocytes, related to Figure 3.

A: Cellular cholesterol, cholesteryl ester (CE), phosphatydilethanolamine (PE), and triacylglycerol (TAG) quantified with HPTLC, normalized to cellular phosphatidylcholine (PC). **B:** Secreted cholesterol, cholesteryl ester (CE) and phosphatydilethanolamine (PE) quantified with HPTLC, normalized to cellular phosphatidylcholine (PC).

Data represent mean ± SD (*: $p < 0.05$; **: $p < 0.01$; ***: One-way ANOVA with post hoc Tukey's multiple comparisons test). All datapoints (n-numbers) are plotted in each bar graph. Experiments were performed in triplicates.

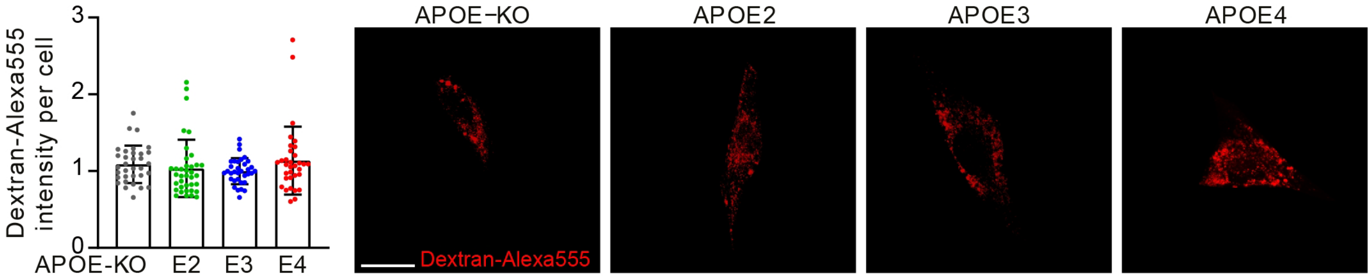

Figure S3. Dextran-Alexa555 uptake does not show genotype-dependent differences, related to Figure 4. Quantification of Dextran-Alexa555 intensity per cell, normalized to APOE3 iAstrocytes, (left) and representative images of Dextran-Alexa555-trested iAstrocytes (right). Scale bar: 50µm. All datapoints (n-numbers) are plotted in each bar graph (3 independent experiments)

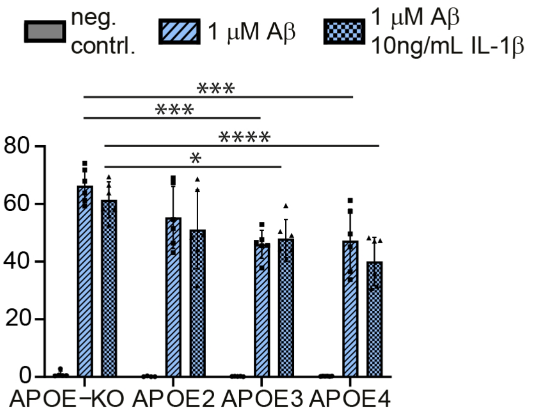

Figure S4. IL-1β activation does not affect allele-dependent Aβ uptake in iAstrocytes, related to Figure 5.

Flow cytometry-based Aβ42 uptake assay showing percentage of Aβ positive cells. Cells were treated with 1μM pre-aggregated Aβ42-hilyte488 alone or pre-treated with 10ng/mL IL-1β. Data represent mean ± SD ***: $p < 0.001$; ****: $p < 0.0001$, Two-way ANOVA with post hoc Holm-Šídák's multiple comparisons test).All datapoints (n-numbers) are plotted in each bar graph (3 independent experiments).

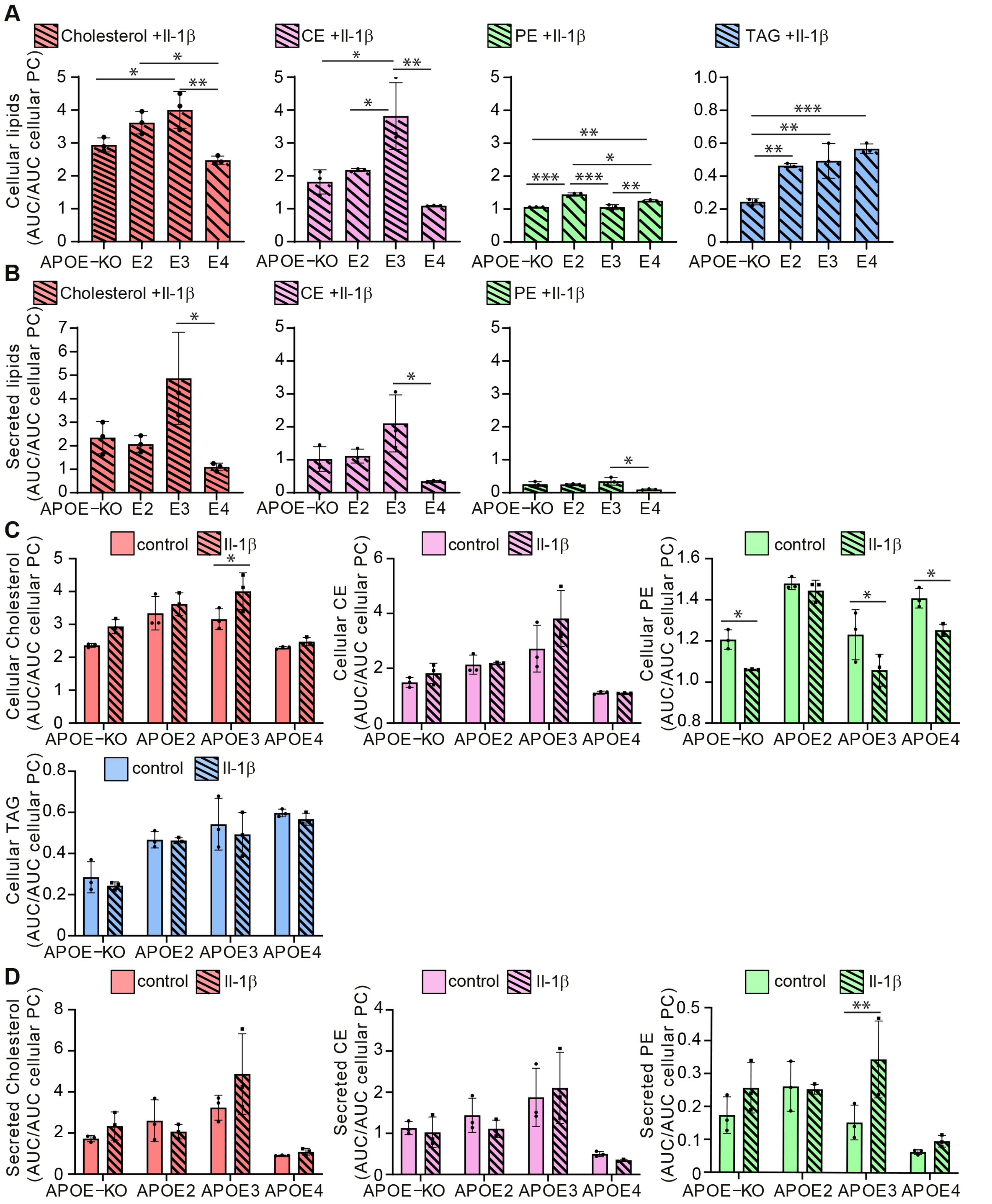

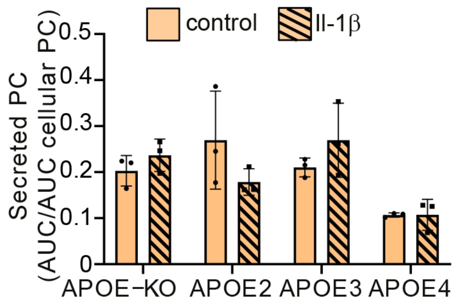

Figure S5. Cholesterol, CE and lipid analysis of the cellular and secreted fractions of IL-1β-treated iAstrocytes, related to Figure 6. A: Bar graphs of cellular cholesterol, cholesterol ester (CE), phosphatydilethanolamine (PE) and triacylglycerol (TAG) in iAstrocytes treated with 10ng/mL IL-1β **B:** Bar graphs of cellular cholesterol, cholesterol ester (CE) and phosphatydilethanolamine (PE) in iAstrocytes treated with 10ng/mL IL-1β. **C:** Bar graphs of cellular cholesterol and lipid quantification at baseline and treated with 10ng/mL IL-1β. **D:** Bar graphs of secreted cholesterol and lipid quantification at baseline and after treatment with 10ng/mL IL-1β. Data represent mean ± SD (*: $p < 0.05$; **: $p < 0.01$; ***: $p < 0.001$; ****: $p < 0.0001$, One-way ANOVA with post hoc Tukey's multiple comparisons test (A, B); Two-way ANOVA with post hoc Holm-Šídák's multiple comparisons test (C, D))). All datapoints (n-numbers) are plotted in each bar graph. Experiments were performed in triplicates.

Supplemental Figure Legend

Figure S1. Genotyping and immunocytochemical characterization of APOE-isogenic iAstrocytes, related to Figure 1 **A:** Agarose gel of genotyping PCR using EzWay Direct APOE Genotyping Kit with a schematic representation of the expected band pattern. **B:** Confocal images of iAstrocytes at day 44, stained with antibody against astrocyte marker GFAP. Scale bar: 150µm. **C:** Confocal images of iAstrocytes at day 30, stained with antibody against astrocyte markers S100β and GJA1. **D:** Confocal images of iAstrocytes at day 30, stained with antibody against astrocyte markers S100β and GJA1. Scale bar: 150µm.

Figure S2. Cholesterol, CE and lipid analysis of the cellular and secreted fractions of iAstrocytes, related to Figure 3. **A:** Cellular cholesterol, cholesteryl ester (CE), phosphatydilethanolamine (PE), and triacylglycerol (TAG) quantified with HPTLC, normalized to cellular phosphatidylcholine (PC). **B:** Secreted cholesterol, cholesteryl ester (CE) and phosphatydilethanolamine (PE) quantified with HPTLC, normalized to cellular phosphatidylcholine (PC).

Data represent mean ± SD (*: $p < 0.05$; **: $p < 0.01$; ***: One-way ANOVA with *post hoc* Tukey's multiple comparisons test). All datapoints (n-numbers) are plotted in each bar graph. Experiments were performed in triplicates.

Figure S3. Dextran-Alexa555 uptake does not show genotype-dependent differences, related to Figure 4. Quantification of Dextran-Alexa555 intensity per cell, normalized to APOE3 iAstrocytes, (left) and representative images of Dextran-Alexa555-trested iAstrocytes (right). Scale bar: 50µm. All datapoints (n-numbers) are plotted in each bar graph (3 independent experiments)

Figure S4. IL-1β activation does not affect allele-dependent Aβ uptake in iAstrocytes, related to Figure 5. Flow cytometry-based Aβ42 uptake assay showing percentage of Aβ positive cells. Cells were treated with 1µM pre-aggregated Aβ42-hilyte488 alone or pre-treated with 10ng/mL IL-1β. Data represent mean ± SD ***: $p < 0.001$; ****: $p < 0.0001$, Two-way ANOVA with *post hoc* Holm-Šídák's multiple comparisons test). All datapoints (n-numbers) are plotted in each bar graph (3 independent experiments)

Figure S5. Cholesterol, CE and lipid analysis of the cellular and secreted fractions of IL-1β-treated iAstrocytes, related to Figure 6. **A:** Bar graphs of cellular cholesterol, cholesterol ester (CE), phosphatydilethanolamine (PE) and triacylglycerol (TAG) in iAstrocytes treated with 10ng/mL IL-1β **B:** Bar graphs of cellular cholesterol, cholesterol ester (CE) and phosphatydilethanolamine (PE) in iAstrocytes treated with 10ng/mL IL-1β . **C:** Bar graphs of cellular cholesterol and lipid quantification at baseline and treated with 10ng/mL IL-1β. **D:** Bar graphs of secreted cholesterol and lipid quantification at baseline and after treatment with 10ng/mL IL-1β.

Data represent mean ± SD (*: $p < 0.05$; **: $p < 0.01$; ***: $p < 0.001$; ****: $p < 0.0001$, One-way ANOVA with *post hoc* Tukey's multiple comparisons test (A, B); Two-way ANOVA with *post hoc* Holm-Šídák's multiple comparisons test (C, D))). All datapoints (n-numbers) are plotted in each bar graph. Experiments were performed in triplicates.

Table S1: NES and leading-edge proteins of analyzed pathways. Table showing adjusted p-values, NES and leading-edge proteins incl. EntrezIDs for GSEA data shown.

Table S2: Statistical analysis of MSD. Table with results of statistical analysis and p-values for all comparisons in the MSD analysis of Figure 5D.

Experimental procedures

Protein sample preparation:

Cells were dissociated at day 44-46 and lysed in RIPA buffer (50 mM Tris-HCL pH7.6, 150 mM NaCl, 1% NP40, 0.5% sodium dodecyl sulfate, 0.5% sodium deoxycholate, and 2mM EDTA (Sigma)) supplemented with cOmplete™ Protease Inhibitor Cocktail (Roche). Lysates were incubated on ice for 20min, followed by sonication and centrifugation (Eppendorf), at 10'000 x g for 10min at 4°C. Total protein concentration was determined using a Pierce BCA Protein Assay Kit (ThermoScientific).

Western blot:

Protein samples were loaded onto a Novex™ 10 to 20%, Tricine, 1.0 mm, Mini Protein Gel (Thermo fisher), and transferred semi-dry onto a Mini 0.2µm Nitrocellulose blot (Biorad). Blots were blocked with 5% non-fat milk (AppliChem) in PBS, and subsequently stained with primary antibody overnight in 5% non-fat Milk in PBS + 0.05% Tween 20 (Sigma). The following primary antibodies were used: FDFT1 (Abcam, ab195046, 1:2000), ABCA1 (Abcam, ab18180, 1:500), HMG-CoA Reductase (Merck-Millipore, ABS229, 1:700), LRP1 (Abcam, EPR3724, 1:1500), VLDLR (Novus, NBP1-78162, 1:500), S100α6 (Abcam, ab181975, 1:1000), α-tubulin (Sigma, T9026, 1:1000), β-actin (Abcam, ab6276, 1:20'000), GJA1 (Abcam, ab235585, 1:1000), Rab5 (Abcam, ab218624, 1:1000), Lamp2 (Biolegend, 354302, 1:500). Blots were subsequently incubated with peroxidase-labeled secondary antibody (anti-goat; 705-036-147, anti-mouse; 715-035-151, anti-rabbit; 111-035-144, Jackson ImmunoResearch, 1:5000), and developed with ECL Prime or Select (Amersham), or Pierce™ ECL (ThermoScientific).

Glutamate uptake assay:

Astrocytes were seeded onto a 20µM fibronectin-coated 96-well plate at previously described density. After 24hr, medium was changed with DMEM without glutamine (Gibco), for 6hr. Subsequently, cells were washed with HEPES buffer (25mM HEPES, 140mM NaCl, 3.5mM KCl, 2.5mM $CaCl_2$, 1mM $MgCl_2$, 0.1% BSA, pH 7.5; Sigma), and incubated for 60min with 20µM glutamate (Sigma) at 37°C. After incubation the residual glutamate was transferred to a white Nunc™ F96 MicroWell™ Polystyrene Plate (ThermoScientific), and kept on ice. The cells were washed with PBS and directly lysed in RIPA buffer, following 20min incubation on ice. During this time the Glutamate Detection Reaction from the Glutamate-Glo assay (Promega) was added to the residual glutamate, and incubated for 60min at room temperature according to protocol. At this time the cell lysates were centrifuged for 15min at 4000 x g at 4°C, following total protein measurement using the Pierce BCA Protein Assay Kit (ThermoScientific). Residual glutamate was measured with luminescence using a TECAN Infinite M1000 plate reader. Glutamate uptake was measured by subtracting the residual glutamate from the positive control, and normalized by the total protein content.

Mass spectrometry proteomic analysis:

Astrocytes were replated to 24-well plates, 24hrs prior to treatment. Cells were treated as mentioned in the previous paragraph, and cells were lysed in RIPA buffer as described. Samples were further processed by using a commercial iST Kit (PreOmics, Germany) with an updated version of the protocol. Briefly, 100 µg of proteins were solubilized in 'Lyse' buffer, boiled at 95°C for 10min and processed with High Intensity Focused Ultrasound (HIFU) for 30s setting the ultrasonic amplitude to 85%. The samples were digested by adding 10µl of the 'Digest' solution. After 60 min of incubation at 37°C the digestion was stopped with 100µl of Stop solution. The solution was transferred to the cartridge and were removed by centrifugation at 3800 x g, while the peptides were retained by the iST-filter. Finally, the peptides were washed, eluted, dried and re-solubilized in 20µl of 3% acetonitrile, 0.1% FA. iRT peptides (Biognosys) were added to each vial.

Mass spectrometry analysis was performed from three biological replicates for each condition on an Orbitrap Fusion Lumos (Thermo Scientific) equipped with a Digital PicoView source (New Objective) and coupled to a M-Class UPLC (Waters). Solvent composition at the two channels was 0.1% formic

acid for channel A and 0.1% formic acid, 99.9% acetonitrile for channel B. For each sample 2µl of peptides were loaded on a commercial MZ Symmetry C18 Trap Column (100Å, 5µm, 180µm x 20 mm, Waters) followed by nanoEase MZ C18 HSS T3 Column (100Å, 1.8µm, 75µm x 250mm, Waters). The peptides were eluted at a flow rate of 300nl/min by a gradient from 5 to 22% B in 80 min and 32% B in 10 min after an initial hold at 5% B for 3 min. The column was washed with 95% B for 10min and afterwards the column was re-equilibrated to starting conditions for additional 10 min. Samples were acquired in a randomized order. The mass spectrometer was operated in data-dependent mode (DDA) acquiring a full-scan MS spectra (300−1'500 m/z) at a resolution of 120'000 at 200 m/z after accumulation to a target value of 500'000. Data-dependent MS/MS were recorded in the linear ion trap using quadrupole isolation with a window of 0.8 Da and HCD fragmentation with 35% fragmentation energy. The ion trap was operated in rapid scan mode with a target value of 10'000 and a maximum injection time of 50ms. Only precursors with intensity above 5'000 were selected for MS/MS and the maximum cycle time was set to 3 s. Charge state screening was enabled. Singly, unassigned, and charge states higher than seven were rejected. Precursor masses previously selected for MS/MS measurement were excluded from further selection for 20s, and the exclusion window was set at 10ppm. The samples were acquired using internal lock mass calibration on m/z 371.1012 and 445.1200. The mass spectrometry proteomics data were handled using the local laboratory information management system (Türker et al., 2010).

Proteomics data processing and analysis:
The acquired raw MS data were processed by MaxQuant (version 1.6.2.3). We obtained protein identification using the integrated Andromeda search engine. We used the canonical sequence Uniprot FASTA database (organism ID 9606, proteome ID UP000005640) downloaded from uniprot.org in July 2019, concatenated to its reversed decoyed database and common protein contaminants (http://fgcz-proteomics.uzh.ch/fasta/fgcz_9606_reviewed_cnl_20190709.fasta). Carbamidomethylation of cysteine was set as fixed, while methionine oxidation and N-terminal protein acetylation we specified as variable modifications. Enzyme specificity was trypsin/P, allowing a minimal peptide length of 7 amino acids and a maximum of two missed cleavages. For search and label-free quantification (LFQ), we used MaxQuant Orbitrap default settings. The maximum false discovery rate (FDR) was set to 0.01 for peptides and 0.05 for proteins. For LFQ, we specified a 2-minute window for match-between-runs. In the MaxQuant experimental design template, each file is kept separate in the experimental design to obtain individual quantitative values.
We inferred protein intensity estimates from peptide intensity values reported in the MaxQuant generated peptides.txt file. We preprocessed the peptide intensities by removing intensities equal to zero and log2 transforming non-zero intensities. To remove systematic differences between samples, we applied a modified robust z-score transformation that preserves the data's original variability. We obtained protein intensity estimates by fitting Tukey's median polish to the peptide data. To estimate fold changes among conditions, i.e., cell lines or APOE genotypes, we fitted a linear model to each protein, then calculated contrasts, and used the Wald test to obtain p-values. Leveraging the parallel structure of the high throughput experiment, we used experimental Bayes to moderate protein variance estimates and updated the t-statistics and p-values accordingly [Smyth, Gordon K]. The p-values were adjusted for multiple testing, using the Benjamini Hochberg procedure and we obtained false discovery rates (FDR). We used the implementation of these methods available in the R package prolfqua (Wolski et al., 2020).
We performed gene set enrichment analysis using the R/Bioconductor package fgsea (Korotkevich et al., 2021) and used gene sets specified in the molecular signature database (http://www.gsea-msigdb.org/). To apply GSEA to proteomics data, we mapped the uniprot identifiers to Entrez Id's using the UniProt mapping service. We ordered the protein lists using the moderated t-statistics. For cases where several UniProt Id's were mapped to a single Entrez Id, we averaged the t-statistic (Smyth, 2004).

Aβ uptake assay:

Astrocytes were plated onto 24-wells plates, and treated with either 10ng/mL IL-1β, 0.2μM RAP (Molecular Innovations), or 10μM GSK2033 (Tocris), for 24hr. Lyophilized Hilyte488-tagged amyloid-β42 (Aβ42-Hilyte488, Anaspec) was diluted to 100μM and sonicated for 10min, followed by shaking at 300RPM for 24hrs at 4°C. After 24hr, cells were treated with the aforementioned compound, in combination with 1μM Aβ42-Hilyte488 for 2hr. Cells were then washed with PBS and and harvested with Trypsin-EDTA (Gibco), transferred to poly-propylene round bottom tubes (BD Bioscience) and washed with FACS buffer (20% FCS (Gibco), 5mM EDTA pH8, 0.01% NaN3 (Sigma), in HBSS (Gibco)). Cells were treated with 0.025% Trypan blue (Gibco) immediately before acquisition of the cells with the LSRII Fortessa 4L (BD Bioscience). Cells for example images were incubated with recombinant Aβ42 (rPeptide), and treated as mentioned before. Cells were incubated with 1μM Aβ42 peptide for 24hrs and fixed according to subchapter "immunocytochemistry".

Lipid extraction:

Lipids from cell pellets and supernatants were extracted using a Bligh and Dyer protocol (Bligh and Dyer, 1959). Briefly, lipids were extracted by adding 3.8mL methanol:chloroform 2:1 (v:v) to a first Wheaton glass tube, followed by 1mL of sample: cell supernatant or cell pellet resuspended in HPLC-grade water. The tube was vortexed thoroughly. Then 1mL chloroform and 1mL water were added, followed by vortexing thoroughly after each addition and centrifugation at 2,000 rpm for 2min at 4°C. The lower phase was transferred to a second Wheaton tube, followed by the addition of 1mL chloroform and 1mL water. In parallel, 1mL chloroform was added to the first Wheaton tube and both tubes were vortexed thoroughly and centrifuged at 2000RPM for 2min at 4°C. The lower phase of the second Wheaton tube was transferred to a final Wheaton tube. The lower phase of the first Wheaton tube was transferred to the second Wheaton tube, vortexed thoroughly and centrifuged at 2000RPM for 2min at 4°C. The lower phase was added to the final Wheaton tube. The solvent was evaporated under vacuum at room temperature. Dried lipids were overlaid with argon and stored at -20°C until further analysis.

High-performance thin-layer chromatography (HPTLC):

Lipids were spotted on HPTLC Silica Gel60 plates (Merck) using an automated TLC sampler (CAMAG ATS 4). The TLC plate was pre-washed in chloroform:methanol (1:1) and dried in a vacuum chamber. For neutral lipids separation and identification, a mix of lipid standards was used: 0.1μg cholesterol (Sigma), 0.1μg cholesteryl ester (Sigma, C9253), 1μg sphingomyelin (Sigma), 1μg phosphatidylcholine (Avanti, 850375), 1μg phosphatidylethanolamine (Avanti, 850725), 1μg triacylglycerol (Sigma). The lipid standard mix and conditioned media/cell pellets lipid extracts resuspended in chloroform:methanol:water (20:9:1) were spotted on the plate using the automated system. The plate was developed first in chloroform:methanol:ammonium hydroxide (65:25:4) for 5cm, dried briefly and re-developed in hexane:diethyl ether:acetic acid (80:20:2) for 9cm. After separation, the HPTLC plate was dried in a vacuum chamber for 30min. Lipids were visualized using a method adapted from Churchward and colleagues (Churchward et al., 2008). Briefly, a copper (II) sulfate staining solution was prepared: 5g of copper (II) sulfate (Sigma, 12849) dissolved in 40mL HPLC-grade water, filtered, mixed with 4.7mL of 85% ortho-phosphoric acid (Merck) and filled up to 50mL with HPLC-grade water. 10mL of freshly prepared staining solution was poured on the HPTLC plate, incubated for 1min, decanted and the plate was dried in the vacuum chamber for 15min. Lipids were then charred at 145°C for 7.5min. The plate was visualised in visible light, at 488nm and 546nm (ChemiDoc MP, Bio-Rad). Lipid spots were quantified using densitometry with the ImageJ software. Lipid content was normalized to cellular phosphatidylcholine content for each sample.

Lysosomal activity assay

Cells were plated and harvested according to "abeta uptake assay", and lysosomal intracellular activity was measured using flow cytometry-based Lysosomal Intracellular Activity Assay Kit (Abcam) according to the manufacturer's protocol.

NFκb P65 transcription factor activity assay

Astrocytes were plated on 12 wells plates as previously described 24hrs prior to treatment with IL1beta or control. Cells were harvested with TE as previously described, and the pellets were reconstituted in 500uL Dynabeads buffer (140mM KCL, 20mM Hepes, 10mM NaCl, 5% glycerol, 2mM MgSO4, 1% Triton X-100, 2mM phenantrolen). Samples were vortexed and gently agitated for 20min at 4C, followed by another round of vortexing. Subsequently samples were centrifuged at 4C for 15min 3000RPM, after which supernatant was removed (the cytosolic and membrane fraction). The pellet was reconstituted in 400uL of dynabeads buffer and spun down at 4C for 5min 3000RPM. Supernatant was discarded and pellets were reconstituted in 100uL RIPA buffer, followed by vortexing. The solution was passed through a 22G needle 10x, and sun down for 30s at 4C at max speed. The supernatant, containing the nuclear fraction, was transferred to a fresh tube and kept at -20C. The NFkB P65 transcription factor activity was measured using the kit from Abcam (ab133112), according to the manufacturer's instructions.

References

Bligh, E.G., and Dyer, W.J. (1959). A rapid method of total lipid extraction and purification. Canadian journal of biochemistry and physiology *37*, 911-917. 10.1139/o59-099.

Churchward, M.A., Brandman, D.M., Rogasevskaia, T., and Coorssen, J.R. (2008). Copper (II) sulfate charring for high sensitivity on-plate fluorescent detection of lipids and sterols: quantitative analyses of the composition of functional secretory vesicles. Journal of chemical biology *1*, 79-87. 10.1007/s12154-008-0007-1.

Korotkevich, G., Sukhov, V., Budin, N., Shpak, B., Artyomov, M.N., and Sergushichev, A. (2021). Fast gene set enrichment analysis. bioRxiv, 060012. 10.1101/060012.

Smyth, G.K. (2004). Linear models and empirical bayes methods for assessing differential expression in microarray experiments. Statistical applications in genetics and molecular biology *3*, Article3. 10.2202/1544-6115.1027.

Türker, C., Akal, F., Joho, D., Panse, C., Barkow-Oesterreicher, S., Rehrauer, H., and Schlapbach, R. (2010). B-Fabric: the Swiss Army Knife for life sciences. Proceedings of the 13th International Conference on Extending Database Technology. Association for Computing Machinery.

Wolski, W., Panse, C., Grossmann, J., D'Errico, M., and Nanni, P. (2020). prolfqua – an R package for proteomics label-free quantification. F1000 Research. doi.org/10.7490/f1000research.1118455.1.
